# Supplementary material for: Transcriptomic Analysis Provides Insight into the ROS Scavenging System and Regulatory Mechanisms in Atriplex canescens Response to Salinity
Source: Int J Mol Sci. 2022 Dec 23;24(1):242. doi: 10.3390/ijms24010242 (PMC9820716; doi:10.3390/ijms24010242)
Supplement: Supplementary file 1 [file ijms-24-00242-s001.zip › ijms-2035176-supplementary.pdf]

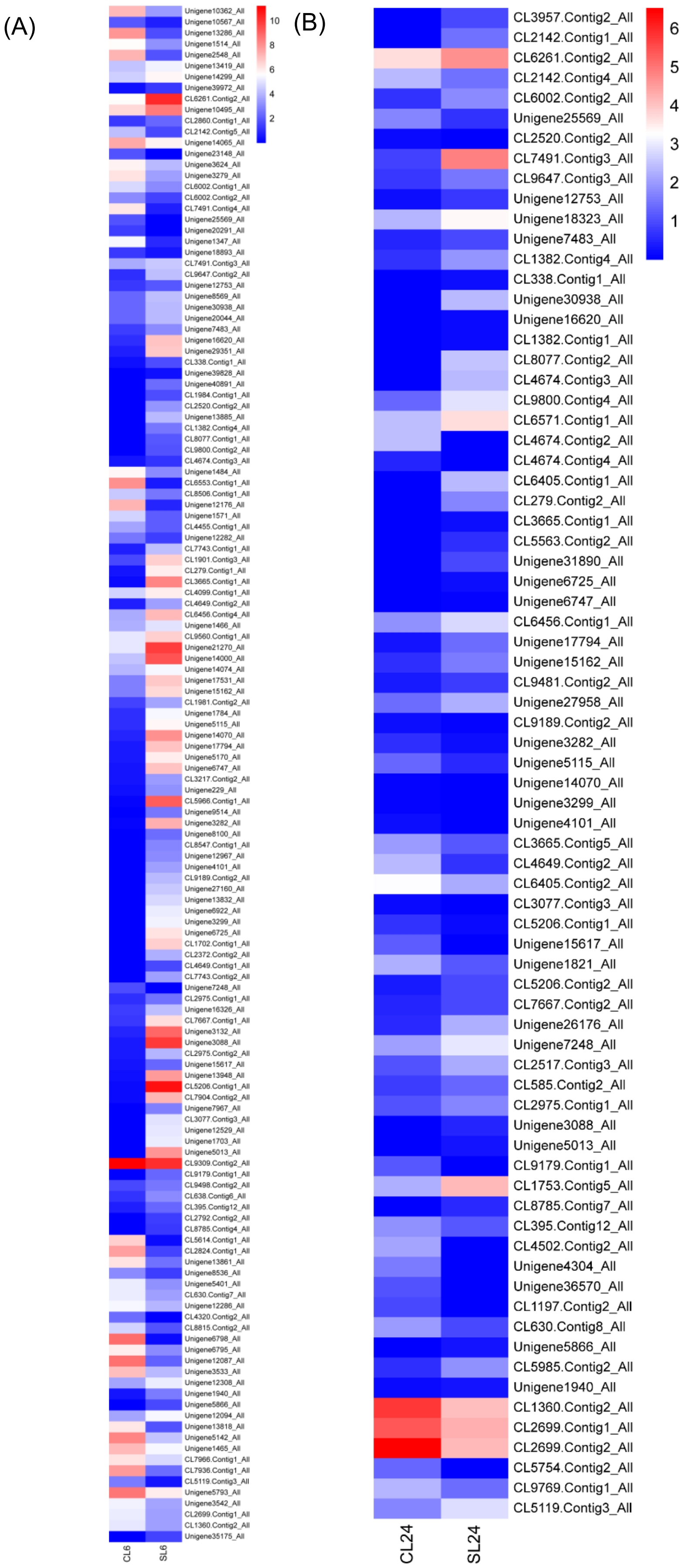

Figure S1. All the DEGs related to the ROS-scavenging system in leaves at (A) 6 h and (B) 24 h under 100 mM NaCl treatment.

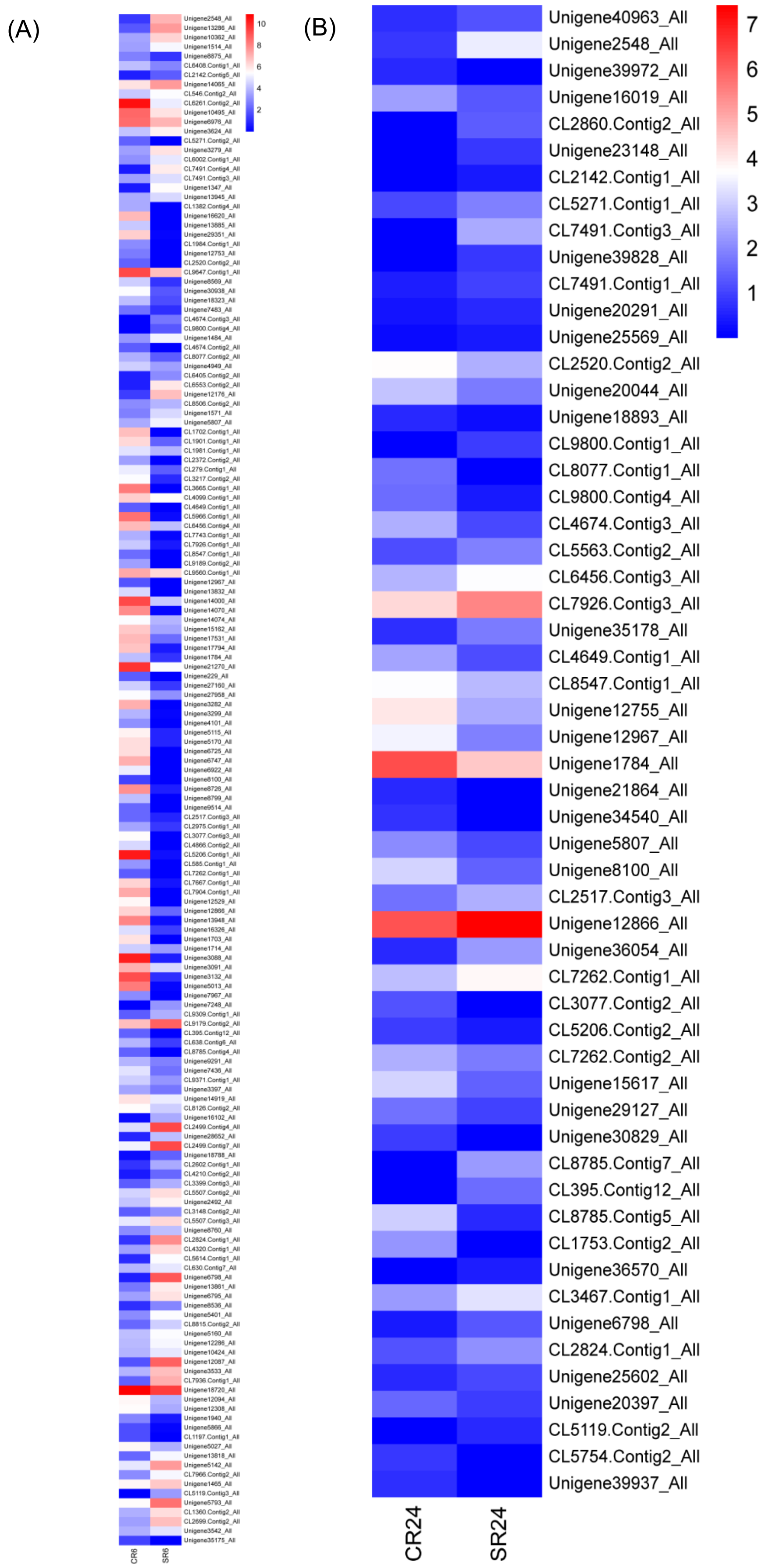

Figure S2. All the DEGs related to the ROS-scavenging system in roots at (A)6 h and (B)24 h under 100 mM NaCl treatment.

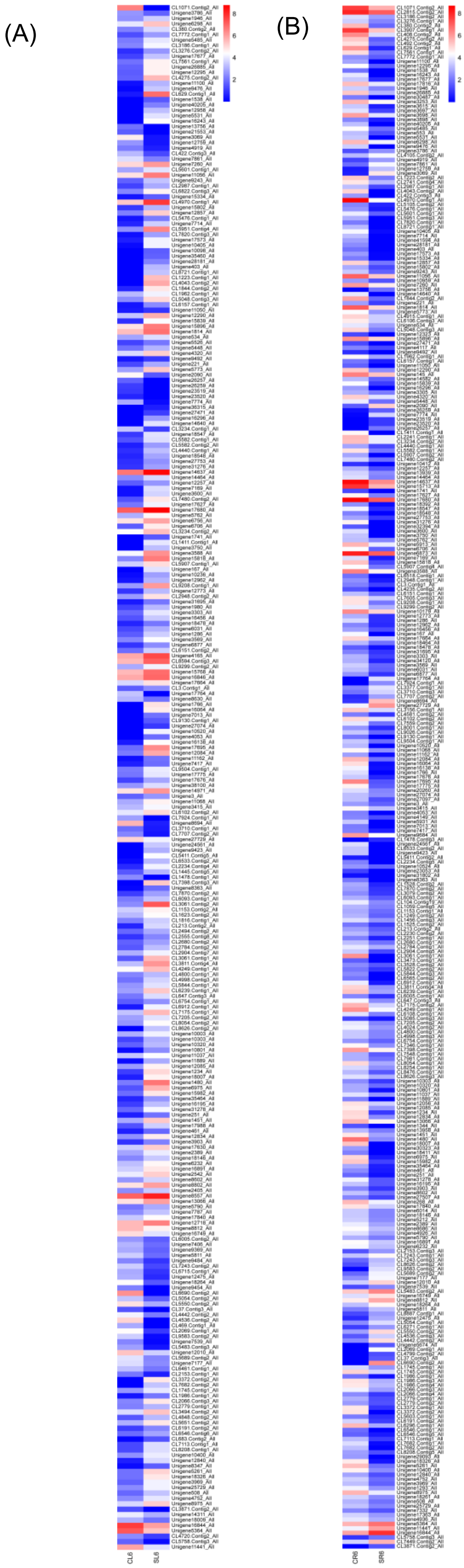

Figure S3. All the DEGs related to transcription factors in (A)leaves and (B)roots at 6 h under 100 mM NaCl treatment.

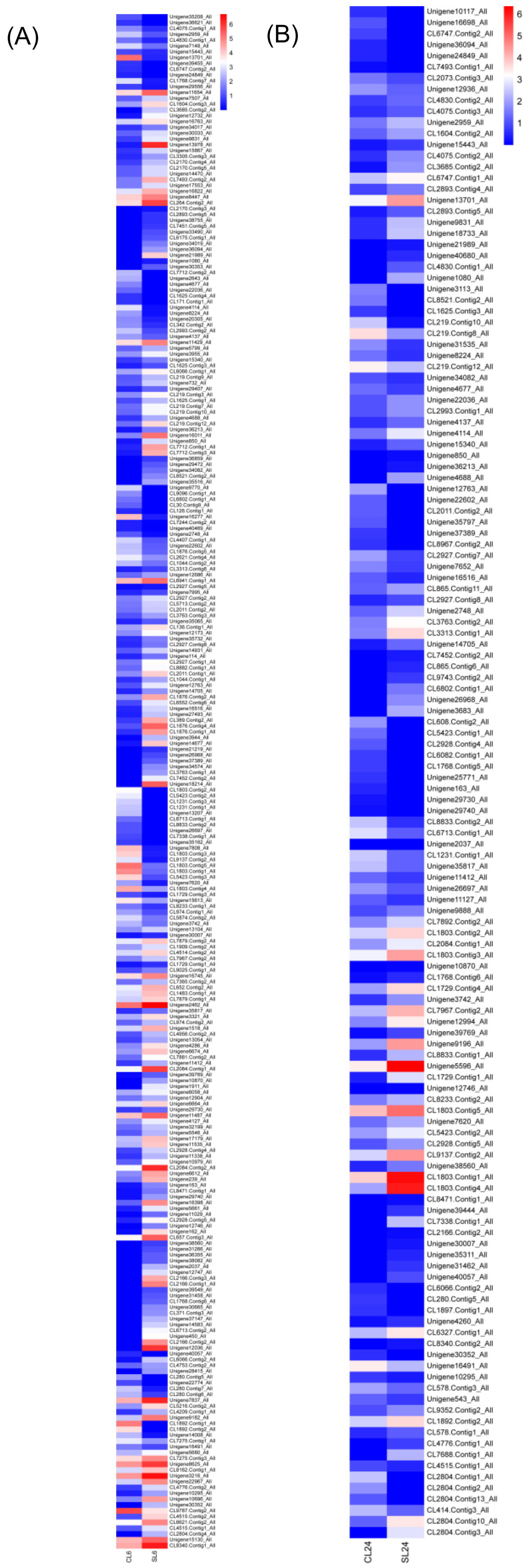

Figure S4. All the DEGs related to protein kinases in leaves at (A)6 h and (B)24 h under 100 mM NaCl treatment.

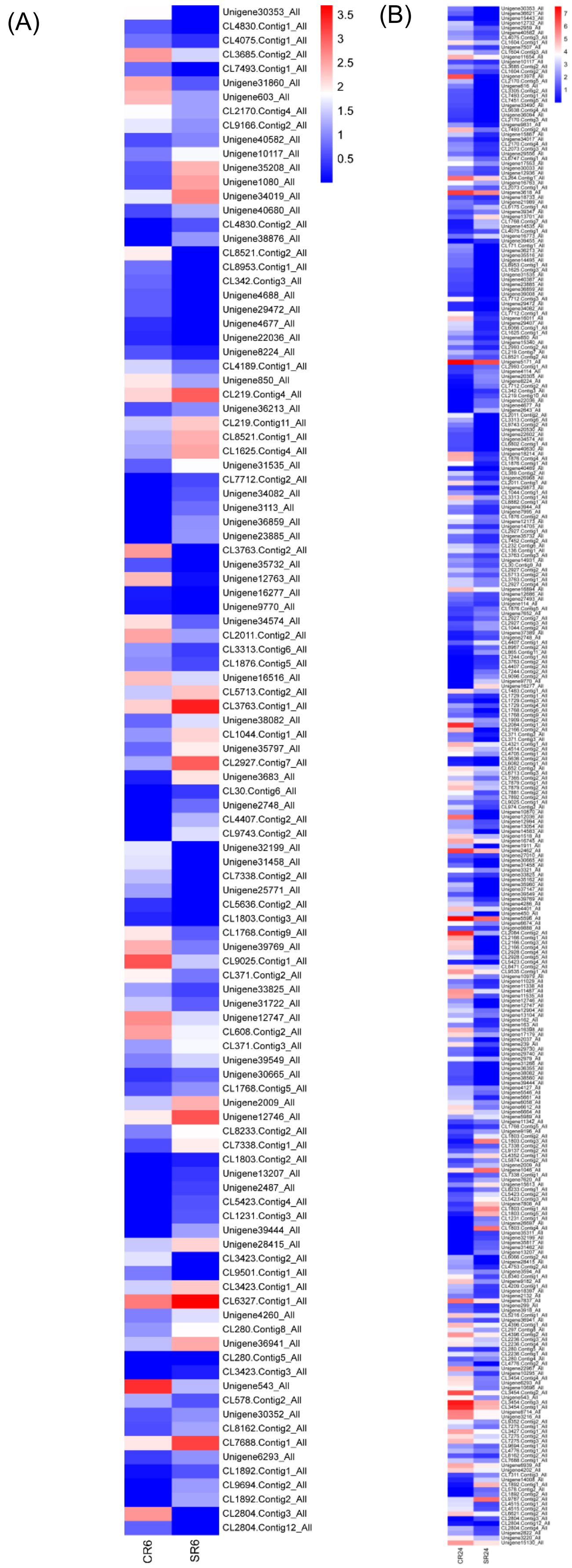

Figure S5. All the DEGs related to protein kinases in roots at (A)6 h and (B)24 h under 100 mM NaCl treatment.

Table S1. The GO enrichment of differentially expressed genes in CL6_vs_SL6.

GO_accession	Description	Ontology	Gene Number
GO:0050896	response to stimulus	BP	340
GO:0055114	oxidation-reduction process	BP	297
GO:0006082	organic acid metabolic process	BP	162
GO:0043436	oxoacid metabolic process	BP	162
GO:0019752	carboxylic acid metabolic process	BP	155
GO:0005975	carbohydrate metabolic process	BP	142
GO:0006629	lipid metabolic process	BP	125
GO:0042221	response to chemical	BP	125
GO:0006520	cellular amino acid metabolic process	BP	89
GO:1901605	alpha-amino acid metabolic process	BP	59
GO:1901700	response to oxygen-containing compound	BP	56
GO:0006979	response to oxidative stress	BP	34
GO:0001101	response to acid chemical	BP	33
GO:0006073	cellular glucan metabolic process	BP	31
GO:0044042	glucan metabolic process	BP	31
GO:0016020	membrane	CC	604
GO:0044425	membrane part	CC	308
GO:0031224	intrinsic component of membrane	CC	269
GO:0016021	integral component of membrane	CC	262
GO:0009536	plastid	CC	216
GO:0009507	chloroplast	CC	208
GO:0044434	chloroplast part	CC	101
GO:0044435	plastid part	CC	101
GO:0031984	organelle subcompartment	CC	73
GO:0009579	thylakoid	CC	72
GO:0005576	extracellular region	CC	66
GO:0044436	thylakoid part	CC	63
GO:0034357	photosynthetic membrane	CC	56
GO:0009534	chloroplast thylakoid	CC	52
GO:0031976	plastid thylakoid	CC	52
GO:0003824	catalytic activity	MF	1635
GO:0016740	transferase activity	MF	638
GO:0016491	oxidoreductase activity	MF	393
GO:0048037	cofactor binding	MF	195
GO:0016757	transferase activity, transferring glycosyl groups	MF	122
GO:0050662	coenzyme binding	MF	122
GO:0016798	hydrolase activity, acting on glycosyl bonds	MF	80
GO:0016758	transferase activity, transferring hexosyl groups	MF	72
GO:0016829	lyase activity	MF	69
GO:0004553	hydrolase activity, hydrolyzing O-glycosyl compounds	MF	68
GO:0016705	oxidoreductase activity, acting on paired donors,	MF	58

	with incorporation or reduction of molecular oxygen		
GO:0046906	tetrapyrrole binding	MF	53
GO:0005506	iron ion binding	MF	52
GO:0020037	heme binding	MF	50
GO:0019842	vitamin binding	MF	47

Table S2. The GO enrichment of differentially expressed genes in CR6_vs_SR6.

GO_accession	Description	Ontology	fg
GO:0005975	carbohydrate metabolic process	BP	155
GO:0009628	response to abiotic stimulus	BP	102
GO:1901700	response to oxygen-containing compound	BP	58
GO:1901605	alpha-amino acid metabolic process	BP	55
GO:0005976	polysaccharide metabolic process	BP	45
GO:0009266	response to temperature stimulus	BP	40
GO:0044264	cellular polysaccharide metabolic process	BP	39
GO:0006979	response to oxidative stress	BP	37
GO:0006325	chromatin organization	BP	35
GO:0006073	cellular glucan metabolic process	BP	34
GO:0044042	glucan metabolic process	BP	34
GO:0006720	isoprenoid metabolic process	BP	29
GO:0016311	dephosphorylation	BP	29
GO:0008299	isoprenoid biosynthetic process	BP	28
GO:0000271	polysaccharide biosynthetic process	BP	27
GO:0016020	membrane	CC	659
GO:0031224	intrinsic component of membrane	CC	292
GO:0016021	integral component of membrane	CC	285
GO:0009536	plastid	CC	224
GO:0009507	chloroplast	CC	214
GO:0044434	chloroplast part	CC	96
GO:0031984	organelle subcompartment	CC	82
GO:0009579	thylakoid	CC	78
GO:0005576	extracellular region	CC	70
GO:0044436	thylakoid part	CC	69
GO:0034357	photosynthetic membrane	CC	63
GO:0009534	chloroplast thylakoid	CC	58
GO:0031976	plastid thylakoid	CC	58
GO:0042651	thylakoid membrane	CC	53
GO:0005618	cell wall	CC	48
GO:0003824	catalytic activity	MF	1706
GO:0016491	oxidoreductase activity	MF	396
GO:0048037	cofactor binding	MF	193
GO:0016757	transferase activity, transferring glycosyl groups	MF	122
GO:0050662	coenzyme binding	MF	119
GO:0016798	hydrolase activity, acting on glycosyl bonds	MF	85

GO:0004553	hydrolase activity, hydrolyzing O-glycosyl compounds	MF	72
GO:0016758	transferase activity, transferring hexosyl groups	MF	72
GO:0016829	lyase activity	MF	66
GO:0016705	oxidoreductase activity, acting on paired donors, with incorporation or reduction of molecular oxygen	MF	60
GO:0046906	tetrapyrrole binding	MF	55
GO:0020037	heme binding	MF	50
GO:0019843	rRNA binding	MF	28
GO:0016209	antioxidant activity	MF	27
GO:0004601	peroxidase activity	MF	25

Table S3. The KEGG pathway enrichment of differentially expressed genes in CL6_vs_SL6.

PathwayID	PathwayName	Gene Number	Pvalue	Qvalue
ko00940	Phenylpropanoid biosynthesis	230	3.95E-09	1.38E-06
ko00945	Stilbenoid, diarylheptanoid and gingerol biosynthesis	103	9.17E-07	0.00016052
ko00860	Porphyrin and chlorophyll metabolism	94	1.60E-05	0.001477621
ko04075	Plant hormone signal transduction	276	1.69E-05	0.001477621
ko00710	Carbon fixation in photosynthetic organisms	110	6.17E-05	0.003599562
ko00941	Flavonoid biosynthesis	104	0.000264463	0.011570274
ko00906	Carotenoid biosynthesis	51	0.000807044	0.025678658
ko01200	Carbon metabolism	261	0.001028398	0.029994928
ko00500	Starch and sucrose metabolism	274	0.001686271	0.042156768
ko00196	Photosynthesis - antenna proteins	26	0.002163035	0.050470827
ko00903	Limonene and pinene degradation	58	0.003684214	0.075851459
ko00480	Glutathione metabolism	80	0.004557604	0.088620081
ko04712	Circadian rhythm - plant	99	0.005864397	0.108028372
ko00195	Photosynthesis	67	0.009469331	0.157822179
ko00511	Other glycan degradation	83	0.013951401	0.221954106
ko00900	Terpenoid backbone biosynthesis	63	0.018697463	0.272671329
ko00030	Pentose phosphate pathway	85	0.021849277	0.298529488
ko00073	Cutin, suberine and wax biosynthesis	52	0.025200117	0.298529488
ko00620	Pyruvate metabolism	92	0.025290438	0.298529488
ko00051	Fructose and mannose metabolism	77	0.025588242	0.298529488
ko00908	Zeatin biosynthesis	39	0.033570703	0.356052913
ko00564	Glycerophospholipid metabolism	105	0.035805157	0.368582494
ko00052	Galactose metabolism	92	0.040431366	0.393082723
ko00010	Glycolysis / Gluconeogenesis	132	0.046668281	0.441456716
ko00350	Tyrosine metabolism	56	0.051016786	0.464955361
ko00590	Arachidonic acid metabolism	22	0.063531515	0.517116982
ko00592	alpha-Linolenic acid metabolism	57	0.075169347	0.571940681
ko00905	Brassinosteroid biosynthesis	15	0.100776372	0.678302507

ko00942	Anthocyanin biosynthesis	17	0.103352623	0.682517324
ko00562	Inositol phosphate metabolism	79	0.112710229	0.704438931

Table S4. The KEGG pathway enrichment of differentially expressed genes in CR6_vs_SR6.

PathwayID	PathwayName	Gene Number	Pvalue	Qvalue
ko00940	Phenylpropanoid biosynthesis	229	1.72E-06	0.00059873
ko00860	Porphyrin and chlorophyll metabolism	98	5.89E-05	0.007820286
ko00945	Stilbenoid, diarylheptanoid and gingerol biosynthesis	100	6.74E-05	0.007820286
ko04075	Plant hormone signal transduction	274	0.002466794	0.12283654
ko00906	Carotenoid biosynthesis	52	0.002654937	0.12283654
ko00710	Carbon fixation in photosynthetic organisms	106	0.002886418	0.12283654
ko00196	Photosynthesis - antenna proteins	27	0.003529786	0.12283654
ko00941	Flavonoid biosynthesis	102	0.005144792	0.162762495
ko00480	Glutathione metabolism	85	0.007302515	0.211772933
ko00500	Starch and sucrose metabolism	287	0.009379824	0.222213807
ko00195	Photosynthesis	72	0.011343157	0.246713668
ko00908	Zeatin biosynthesis	45	0.014097839	0.288591065
ko00052	Galactose metabolism	105	0.015247098	0.294777224
ko00592	alpha-Linolenic acid metabolism	68	0.018431396	0.337585566
ko04712	Circadian rhythm - plant	102	0.020181192	0.341488187
ko00900	Terpenoid backbone biosynthesis	68	0.020607046	0.341488187
ko00564	Glycerophospholipid metabolism	117	0.021802754	0.344879931
ko00511	Other glycan degradation	86	0.035052676	0.469166584
ko00350	Tyrosine metabolism	62	0.039102834	0.503992089
ko00360	Phenylalanine metabolism	55	0.050588085	0.535741933
ko01200	Carbon metabolism	256	0.055756896	0.554382849
ko00073	Cutin, suberine and wax biosynthesis	52	0.076172257	0.602453309
ko00903	Limonene and pinene degradation	52	0.076172257	0.602453309
ko00750	Vitamin B6 metabolism	20	0.0801563	0.603903147
ko00740	Riboflavin metabolism	20	0.0801563	0.603903147
ko00590	Arachidonic acid metabolism	23	0.084066824	0.603903147
ko00950	Isoquinoline alkaloid biosynthesis	44	0.08503234	0.603903147
ko00030	Pentose phosphate pathway	85	0.091519952	0.618508588
ko00010	Glycolysis / Gluconeogenesis	139	0.093297084	0.618508588
ko00770	Pantothenate and CoA biosynthesis	43	0.097680617	0.618508588

Table S5. The GO enrichment of differentially expressed genes in CL24_vs_SL24.

GO_accession	Description	Ontology	Gene Number
GO:0050896	response to stimulus	BP	84
GO:0006796	phosphate-containing compound metabolic process	BP	73
GO:0006793	phosphorus metabolic process	BP	73
GO:0043412	macromolecule modification	BP	64
GO:0065007	biological regulation	BP	62
GO:0016310	phosphorylation	BP	59
GO:0050789	regulation of biological process	BP	56
GO:0006464	cellular protein modification process	BP	55
GO:0036211	protein modification process	BP	55
GO:0050794	regulation of cellular process	BP	54
GO:0051716	cellular response to stimulus	BP	44
GO:0006468	protein phosphorylation	BP	43
GO:0007154	cell communication	BP	42
GO:0007165	signal transduction	BP	38
GO:0023052	signaling	BP	38
GO:0016020	membrane	CC	113
GO:0044425	membrane part	CC	68
GO:0016021	integral component of membrane	CC	61
GO:0031224	intrinsic component of membrane	CC	61
GO:0012505	endomembrane system	CC	22
GO:0005794	Golgi apparatus	CC	11
GO:0098791	Golgi subcompartment	CC	8
GO:0044431	Golgi apparatus part	CC	8
GO:0000139	Golgi membrane	CC	7
GO:0016604	nuclear body	CC	6
GO:0000786	nucleosome	CC	4
GO:0032993	protein-DNA complex	CC	4
GO:0044815	DNA packaging complex	CC	4
GO:0009514	glyoxysome	CC	3
GO:0031985	Golgi cisterna	CC	3
GO:0016740	transferase activity	MF	146
GO:0016772	transferase activity, transferring phosphorus-containing groups	MF	79
GO:0008144	drug binding	MF	79
GO:0016301	kinase activity	MF	69
GO:0016773	phosphotransferase activity, alcohol group as acceptor	MF	66
GO:0004672	protein kinase activity	MF	56
GO:0004674	protein serine/threonine kinase activity	MF	37
GO:0016758	transferase activity, transferring hexosyl groups	MF	19
GO:0005509	calcium ion binding	MF	15
GO:0046983	protein dimerization activity	MF	11
GO:0046527	glucosyltransferase activity	MF	10

GO:0008194	UDP-glycosyltransferase activity	MF	9
GO:0004601	peroxidase activity	MF	8
GO:0016684	oxidoreductase activity, acting on peroxide as acceptor	MF	8
GO:0016209	antioxidant activity	MF	8

Table S6. The GO enrichment of differentially expressed genes in CR24_vs_SR24.

GO_accession	Description	Ontology	Gene Number
GO:0008152	metabolic process	BP	288
GO:0009987	cellular process	BP	247
GO:0044237	cellular metabolic process	BP	224
GO:0071704	organic substance metabolic process	BP	214
GO:0044238	primary metabolic process	BP	201
GO:0006807	nitrogen compound metabolic process	BP	174
GO:0043170	macromolecule metabolic process	BP	147
GO:0009058	biosynthetic process	BP	141
GO:1901564	organonitrogen compound metabolic process	BP	140
GO:0034641	cellular nitrogen compound metabolic process	BP	136
GO:0044260	cellular macromolecule metabolic process	BP	135
GO:1901576	organic substance biosynthetic process	BP	133
GO:0044249	cellular biosynthetic process	BP	131
GO:0044271	cellular nitrogen compound biosynthetic process	BP	112
GO:0019538	protein metabolic process	BP	109
GO:0005622	intracellular	CC	188
GO:0044424	intracellular part	CC	183
GO:0043229	intracellular organelle	CC	156
GO:0043226	organelle	CC	156
GO:0005737	cytoplasm	CC	152
GO:0044444	cytoplasmic part	CC	131
GO:0032991	protein-containing complex	CC	96
GO:0043228	non-membrane-bounded organelle	CC	78
GO:0043232	intracellular non-membrane-bounded organelle	CC	78
GO:0005840	ribosome	CC	73
GO:1990904	ribonucleoprotein complex	CC	73
GO:0009534	chloroplast thylakoid	CC	13
GO:0031976	plastid thylakoid	CC	13
GO:0044391	ribosomal subunit	CC	13
GO:0009521	photosystem	CC	7
GO:0005198	structural molecule activity	MF	68
GO:0003735	structural constituent of ribosome	MF	67
GO:0016829	lyase activity	MF	19
GO:0016830	carbon-carbon lyase activity	MF	12
GO:0016407	acetyltransferase activity	MF	7
GO:0008374	O-acyltransferase activity	MF	6
GO:0004527	exonuclease activity	MF	5

GO:0016833	oxo-acid-lyase activity	MF	4
GO:0038023	signaling receptor activity	MF	4
GO:0003993	acid phosphatase activity	MF	4
GO:0009001	serine O-acetyltransferase activity	MF	3
GO:0016412	serine O-acyltransferase activity	MF	3
GO:0016413	O-acetyltransferase activity	MF	3
GO:0003913	DNA photolyase activity	MF	3
GO:0052716	hydroquinone: oxygen oxidoreductase activity	MF	3

Table S7. The KEGG pathway enrichment of differentially expressed genes in CL24_vs_SL24.

PathwayID	PathwayName	Gene Number	Pvalue	Qvalue
ko00531	Glycosaminoglycan degradation	21	0.000164426	0.051133044
ko04146	Peroxisome	34	0.001437022	0.152324343
ko00564	Glycerophospholipid metabolism	32	0.002625411	0.166976156
ko00603	Glycosphingolipid biosynthesis - globo series	8	0.006016992	0.218958538
ko00590	Arachidonic acid metabolism	9	0.00619694	0.218958538
ko00650	Butanoate metabolism	13	0.007416156	0.235833762
ko00860	Porphyrin and chlorophyll metabolism	21	0.00923778	0.267055825
ko00072	Synthesis and degradation of ketone bodies	5	0.010962875	0.272046292
ko00280	Valine, leucine and isoleucine degradation	20	0.011257365	0.272046292
ko02010	ABC transporters	33	0.020723831	0.346851484
ko00941	Flavonoid biosynthesis	23	0.025955724	0.375178194
ko00770	Pantothenate and CoA biosynthesis	12	0.039592307	0.490763338
ko00592	alpha-Linolenic acid metabolism	16	0.04125537	0.490763338
ko04120	Ubiquitin mediated proteolysis	33	0.041668585	0.490763338
ko00290	Valine, leucine and isoleucine biosynthesis	9	0.050039906	0.548175635
ko00730	Thiamine metabolism	5	0.052830815	0.548175635
ko00740	Riboflavin metabolism	6	0.053438505	0.548175635
ko00600	Sphingolipid metabolism	18	0.073476743	0.589639076
ko04070	Phosphatidylinositol signaling system	21	0.074134341	0.589639076
ko00660	C5-Branched dibasic acid metabolism	5	0.081304707	0.601276674
ko00360	Phenylalanine metabolism	12	0.125890338	0.835122115
ko03040	Spliceosome	69	0.131308509	0.835122115
ko00790	Folate biosynthesis	6	0.140929931	0.87873957
ko00561	Glycerolipid metabolism	18	0.153088089	0.901518744
ko04144	Endocytosis	57	0.165427637	0.923416099
ko00510	N-Glycan biosynthesis	11	0.16551798	0.923416099
ko00903	Limonene and pinene degradation	11	0.178830864	0.948526836
ko00440	Phosphonate and phosphinate metabolism	3	0.17890257	0.948526836

ko00400	Phenylalanine, tyrosine and tryptophan biosynthesis	12	0.178967328	0.948526836
ko00591	Linoleic acid metabolism	6	0.200090363	0.999979679

Table S8. The KEGG pathway enrichment of differentially expressed genes in CR24_vs_SR24.

PathwayID	PathwayName	Gene number	Pvalue	Qvalue
ko03010	Ribosome	91	9.34E-14	2.89E-11
ko00196	Photosynthesis - antenna proteins	14	2.72E-08	4.22E-06
ko00860	Porphyrin and chlorophyll metabolism	18	0.001431581	0.11094751
ko00195	Photosynthesis	16	0.002055616	0.11694497
ko00450	Selenocompound metabolism	9	0.003878925	0.171780984
ko00710	Carbon fixation in photosynthetic organisms	19	0.007640534	0.263173938
ko00770	Pantothenate and CoA biosynthesis	10	0.016925359	0.476987396
ko00590	Arachidonic acid metabolism	6	0.022188225	0.555205424
ko03060	Protein export	12	0.023425012	0.555205424
ko03015	mRNA surveillance pathway	36	0.033693407	0.555205424
ko03013	RNA transport	57	0.034145873	0.555205424
ko00790	Folate biosynthesis	6	0.034263224	0.555205424
ko00740	Riboflavin metabolism	5	0.035545105	0.555205424
ko00941	Flavonoid biosynthesis	16	0.042452455	0.598193679
ko02010	ABC transporters	22	0.052630773	0.674419634
ko00072	Synthesis and degradation of ketone bodies	3	0.05438868	0.674419634
ko04120	Ubiquitin mediated proteolysis	23	0.056660284	0.675564928
ko03450	Non-homologous end-joining	3	0.069798124	0.763352093
ko00511	Other glycan degradation	14	0.069800327	0.763352093
ko00510	N-Glycan biosynthesis	9	0.08730129	0.795982348
ko00940	Phenylpropanoid biosynthesis	27	0.093227	0.825724853
ko04122	Sulfur relay system	4	0.103201471	0.864660975
ko00970	Aminoacyl-tRNA biosynthesis	16	0.12524438	0.975200337
ko00600	Sphingolipid metabolism	12	0.128030482	0.975200337
ko04070	Phosphatidylinositol signaling system	14	0.128978109	0.975200337
ko04146	Peroxisome	17	0.144332013	0.991463594
ko01210	2-Oxocarboxylic acid metabolism	15	0.16122143	0.997976318
ko00760	Nicotinate and nicotinamide metabolism	6	0.176157809	0.997976318
ko00562	Inositol phosphate metabolism	13	0.176613206	0.997976318
ko00945	Stilbenoid, diarylheptanoid and gingerol biosynthesis	11	0.184525917	0.997976318

Table S9. Differentially expressed genes (DEGs) related to enzymatic antioxidant defense systems in leaves of *A. canescens* after 100 mM NaCl treatment for 6 h. FPKM-CL6 and FPKM-SL6 respectively indicates the FPKM value of a gene in leaves under control condition for 6 h and salt treatment for 6 h. Fold change equals to log2 (FPKM-SL6 / FPKM-CL6). Protein refer to the protein encoded by each DEGs.

Gene ID	FPKM-CL6	FPKM-SL6	Fold change	*Homologous species*

**GLR**				
Unigene10362_All	138.82	9.94	-3.80	*Prunus mume*
Unigene10567_All	2.88	0.67	-2.10	*Rheum australe*
Unigene13286_All	244.75	2.43	-6.65	*Malus domestica*
Unigene1514_All	51.79	8.1	-2.68	*Prunus mume*
Unigene2548_All	149.71	2.57	-5.86	*Medicago truncatula*
Unigene13419_All	19.38	38.9	1.01	*Fragaria vesca subsp. Vesca*
Unigene14299_All	22.39	55.68	1.31	*Cucumis melo subsp. Melo*
Unigene39972_All	0.29	1.43	2.30	*Ricinus communis*
**APX**				
CL6261.Contig2_All	51.09	1266.04	4.63	*Mesembryanthemum crystallinum*
Unigene10495_All	90.75	358.03	1.98	*Mesembryanthemum crystallinum*
CL2860.Contig1_All	1.38	3.83	1.47	*Nelumbo nucifera*
CL2142.Contig5_All	18.02	2.19	-3.04	*Vitis vinifera*
Unigene14065_All	173.04	59	-1.55	*Beta vulgaris*
Unigene23148_All	2.64	0.01	-8.04	*Theobroma cacao*
**MDAR**				
Unigene3624_All	58.95	16.15	-1.87	*Populus trichocarpa*
**DHAR**				
Unigene3279_All	73.9	10.69	-2.7	*Spinacia oleracea*
**GR**				
CL6002.Contig1_All	26.75	7.67	-1.80	*Dimocarpus longan*
CL6002.Contig2_All	7.83	1.96	-2.00	*Morus notabilis*
**GST**				
CL7491.Contig4_All	69.03	0.7	-6.62	*Salicornia brachiata*
Unigene25569_All	2.68	0.01	-8.07	*Theobroma cacao*
Unigene20291_All	1.77	0.01	-7.47	*Knorringia sibirica*
Unigene1347_All	42.65	0.95	-5.49	*Vitis vinifera*
Unigene18893_All	1.51	0.42	-1.85	*Brassica rapa*
CL7491.Contig3_All	12.91	20.88	0.69	*Salicornia brachiata*
CL9647.Contig2_All	1.06	17.3	4.03	*Dianthus caryophyllus*
Unigene12753_All	1.4	2.87	1.04	*Nelumbo nucifera*
Unigene8569_All	4.14	18.31	2.14	*Cicer arietinum*
Unigene30938_All	4.05	16.31	2.01	*Fragaria vesca subsp. Vesca*
Unigene20044_All	4.04	15.84	1.97	*Solanum lycopersicum*
Unigene7483_All	1.61	7.78	2.27	*[Salicornia brachiata*
Unigene16620_All	1.14	121.94	6.74	*Eucalyptus grandis*
Unigene29351_All	0.74	114.88	7.28	*Eucalyptus grandis*
CL338.Contig1_All	0.37	2.4	2.70	*Nelumbo nucifera*
Unigene39828_All	0.01	0.36	5.17	*Malva pusilla*
Unigene40891_All	0.01	4.42	8.79	*Prunus mume*
CL1984.Contig1_All	0.01	2.25	7.81	*Fragaria vesca subsp. Vesca*
CL2520.Contig2_All	0.01	9.01	9.82	*Vitis vinifera*
Unigene13885_All	0.01	16.44	10.68	*Malus domestica*

CL1382.Contig4_All	0.01	5.16	9.01	*Nicotiana sylvestris*
**GPX**				
CL8077.Contig1_All	0.01	2.87	8.16	*Theobroma cacao*
CL9800.Contig2_All	0.01	2.73	8.09	*Spinacia oleracea*
CL4674.Contig3_All	0.43	1.23	1.52	*Theobroma cacao*
Unigene1484_All	55.66	7.91	-2.81	*Vitis vinifera*
**POD**				
CL6553.Contig1_All	260.56	0.58	-8.81	*Spinacia oleracea*
CL8506.Contig1_All	20.37	5.32	-1.94	*Spinacia oleracea*
Unigene12176_All	158.63	0.75	-7.72	*Spinacia oleracea*
Unigene1571_All	24.22	3.12	-2.96	*Spinacia oleracea*
CL4455.Contig1_All	11.49	3.38	-1.77	*Solanum tuberosum*
Unigene12282_All	5.38	1.68	-1.68	*Citrus sinensis*
CL7743.Contig1_All	0.73	17.99	4.62	*Spinacia oleracea*
CL1901.Contig3_All	2.11	101.61	5.59	*Vitis vinifera*
CL279.Contig1_All	0.42	63.45	7.24	*Fragaria x ananassa*
CL3665.Contig1_All	0.21	315.19	10.55	*Spinacia oleracea*
CL4099.Contig1_All	23.65	65.77	1.48	*Camellia oleifera*
CL4649.Contig2_All	0.62	10.72	4.11	*Vitis vinifera*
CL6456.Contig4_All	12.89	136.7	3.41	*Phoenix dactylifera*
Unigene1466_All	13.92	30.78	1.14	*Spinacia oleracea*
CL9560.Contig1_All	34.42	99.61	1.53	*Phoenix dactylifera*
Unigene21270_All	32.85	935.17	4.83	*Nelumbo nucifera*
Unigene14000_All	19.04	657.68	5.11	*Spinacia oleracea*
Unigene14074_All	15.29	42.06	1.46	*Theobroma cacao*
Unigene17531_All	6.58	109.29	4.05	*Sesuvium portulacastrum*
Unigene15162_All	6.32	87.95	3.80	*Nelumbo nucifera*
CL1981.Contig2_All	1.93	11.59	2.59	*Spinacia oleracea*
Unigene1784_All	1.09	41.53	5.25	*Citrus sinensis*
Unigene5115_All	1.06	54.9	5.69	*Vitis vinifera*
Unigene14070_All	0.75	258.24	8.43	*Spinacia oleracea*
Unigene17794_All	0.6	118.26	7.62	*Spinacia oleracea*
Unigene5170_All	0.55	66.59	6.92	*Nicotiana tabacum*
Unigene6747_All	0.39	121.04	8.28	*Stylosanthes humilis*
CL3217.Contig2_All	0.44	9.62	4.45	*Eucalyptus grandis*
Unigene229_All	0.28	8.55	4.93	*Pyrus x bretschneideri*
CL5966.Contig1_All	0.18	588.89	11.68	*Nelumbo nucifera*
Unigene9514_All	0.09	5.44	5.92	*Fragaria vesca subsp. Vesca*
Unigene3282_All	0.1	159.13	10.64	*Spinacia oleracea*
Unigene8100_All	0.01	4.23	8.72	*Musa acuminata*
CL8547.Contig1_All	0.01	6.96	9.44	*Malus domestica*
Unigene12967_All	0.01	7.65	9.58	*Malus domestica*
Unigene4101_All	0.01	10.33	10.01	*Beta vulgaris*
CL9189.Contig2_All	0.01	15.98	10.64	*Beta vulgaris*

Unigene27160_All	0.01	20.41	11.00	*Nelumbo nucifera*
Unigene13832_All	0.01	25.94	11.34	*Theobroma cacao*
Unigene6922_All	0.01	35.76	11.80	*Pyrus x bretschneideri*
Unigene3299_All	0.01	40.52	11.98	*Spinacia oleracea*
Unigene6725_All	0.01	74.38	12.86	*Beta vulgaris*
CL1702.Contig1_All	0.01	105.77	13.37	*Spinacia oleracea*
CL2372.Contig2_All	0.01	13.4	10.39	*Citrus sinensis*
CL4649.Contig1_All	0.01	2.24	7.81	*Vitis vinifera*
CL7743.Contig2_All	0.01	10.34	10.01	*Spinacia oleracea*
**GLP**				
Unigene7248_All	2.43	0.01	-7.92	*Solanum tuberosum*
CL2975.Contig1_All	1.07	4.86	2.18	*Vitis vinifera*
Unigene16326_All	1.7	17.64	3.38	*Beta vulgaris*
CL7667.Contig1_All	1.39	78.63	5.82	*Atriplex lentiformis*
Unigene3132_All	0.88	475.44	9.08	*Nelumbo nucifera*
Unigene3088_All	0.56	1004.78	10.81	*Atriplex lentiformis*
CL2975.Contig2_All	0.61	15.22	4.64	*Vitis vinifera*
Unigene15617_All	0.43	4.03	3.23	*Solanum lycopersicum*
Unigene13948_All	0.32	229.65	9.49	*Atriplex lentiformis*
CL5206.Contig1_All	0.23	1797.03	12.93	*Glycine max*
CL7904.Contig2_All	0.2	157.39	9.62	*Beta vulgaris*
Unigene7967_All	0.01	6.55	9.36	*Beta vulgaris*
CL3077.Contig3_All	0.01	30.51	11.58	*Prunus mume*
Unigene12529_All	0.01	32.97	11.69	*Beta vulgaris*
Unigene1703_All	0.01	37.74	11.88	*Prunus mume*
Unigene5013_All	0.01	253.97	14.63	*Mesembryanthemum crystallinum*
**CAT**				
CL9309.Contig2_All	2442.03	1163.48	-1.07	*Gardenia jasminoides*
CL9179.Contig1_All	0.01	3.93	8.62	*Suaeda salsa*
**PEX**				
CL9498.Contig2_All	2.1	5.48	1.38	*Vitis vinifera*
CL638.Contig6_All	1.25	7.58	2.60	*Medicago truncatula*
CL395.Contig12_All	0.7	3.95	2.50	*Medicago truncatula*
CL2792.Contig2_All	0.01	1.72	7.43	*Vitis vinifera*
CL8785.Contig4_All	0.01	1.54	7.27	*Theobroma cacao*
**Trx**				
CL5614.Contig1_All	100.83	0.25	-8.66	*Brassica rapa*
CL2824.Contig1_All	215.76	1.8	-6.91	*Spinacia oleracea*
Unigene13861_All	74.44	4.96	-3.91	*Eucalyptus grandis*
Unigene8536_All	7.31	1.65	-2.15	*Theobroma cacao*
Unigene5401_All	36.05	8.42	-2.10	*Eucalyptus grandis*
CL630.Contig7_All	37.99	11.15	-1.77	*Solanum tuberosum*
Unigene12286_All	44.31	15.05	-1.56	*Theobroma cacao*
CL4320.Contig2_All	4.4	0.01	-8.78	*Vitis vinifera*

CL8815.Contig2_All	24.25	2.68	-3.18	*Malus domestica*
Unigene6798_All	464.18	0.22	-11.04	*Spinacia oleracea*
Unigene6795_All	61.94	7.9	-2.97	*Cucumis melo*
Unigene12087_All	429.05	3.72	-6.85	*Spinacia oleracea*
Unigene3533_All	130.41	16.13	-3.02	*Spinacia oleracea*
Unigene12308_All	11.51	37.92	1.72	*Fragaria vesca subsp. Vesca*
Unigene1940_All	0.45	5.92	3.72	*Prunus mume*
Unigene5866_All	0.08	2.13	4.73	*Vitis vinifera*
Unigene12094_All	11.55	44.93	1.96	*Tamarix hispida*
**PrxR**				
Unigene13818_All	71.42	2.76	-4.69	*Vitis vinifera*
Unigene5142_All	315.26	19.53	-4.01	*Tamarix hispida*
Unigene1465_All	145.95	41.76	-1.81	*Tamarix hispida*
CL7966.Contig1_All	76.42	25.89	-1.56	*Cicer arietinum*
CL7936.Contig1_All	218.86	4.31	-5.67	*Suaeda salsa*
**SOD**				
CL5119.Contig3_All	6.05	0.44	-3.78	*Nicotiana tomentosiformis*
Unigene5793_All	405.05	65.24	-2.63	*Phytolacca acinosa*
Unigene3542_All	38.69	11.5	-1.75	*Mesembryanthemum crystallinum*
CL2699.Contig1_All	36.92	10.33	-1.84	*Spinacia oleracea*
CL1360.Contig2_All	33.68	11.18	-1.59	*Chenopodiastrum murale*
Unigene35175_All	0.01	1.91	7.58	*Mesembryanthemum crystallinum*

Table S10. Differentially expressed genes (DEGs) related to enzymatic antioxidant defense systems in leaves of *A. canescens* after 100 mM NaCl treatment for 24 h. FPKM-CL24 and FPKM-SL24 respectively indicates the FPKM value of a gene in leaves under control condition for 24 h and salt treatment for 24 h. Fold change equals to log2 (FPKM-SL24 / FPKM-CL24). Protein refer to the protein encoded by each DEGs.

Gene ID	FPKM-CL24	FPKM-SL24	Fold change	Homologous species
**GLR**				
CL3957.Contig2_All	0.01	0.97	6.60	Rheum australe
**APX**				
CL2142.Contig1_All	0.01	1.83	7.52	Vitis vinifera
CL6261.Contig2_All	12.07	24.51	1.02	Mesembryanthemum crystallinum
CL2142.Contig4_All	4.2	1.8	-1.22	Vitis vinifera
**GR**				
CL6002.Contig2_All	0.62	2.47	1.99	Morus notabilis
**GST**				
Unigene25569_All	2.38	0.64	-1.89	Theobroma cacao
CL2520.Contig2_All	0.11	0.01	-3.46	Vitis vinifera
CL7491.Contig3_All	0.86	28.08	5.03	Salicornia brachiata
CL9647.Contig3_All	0.68	1.95	1.52	Dianthus caryophyllus

Unigene12753_All	0.18	0.72	2.00	Nelumbo nucifera
Unigene18323_All	4.01	9.39	1.23	Nelumbo nucifera
Unigene7483_All	0.42	0.97	1.21	Salicornia brachiata
CL1382.Contig4_All	0.66	2.83	2.10	Solanum tuberosum
CL338.Contig1_All	0.01	0.17	4.09	Nelumbo nucifera
Unigene30938_All	0.01	4.22	8.72	Fragaria vesca subsp. vesca
Unigene16620_All	0.01	0.11	3.46	Eucalyptus grandis
CL1382.Contig1_All	0.01	0.12	3.58	Dianthus caryophyllus
**GSH-Px**				
CL8077.Contig2_All	0.01	4.66	8.86	Theobroma cacao
CL4674.Contig3_All	0.01	4.25	8.73	Theobroma cacao
CL9800.Contig4_All	1.57	6.51	2.05	Spinacia oleracea
CL6571.Contig1_All	4.58	11.98	1.39	Spinacia oleracea
CL4674.Contig2_All	4.46	0.01	-8.80	
CL4674.Contig4_All	0.43	0.01	-5.43	Theobroma cacao
**POD**				
CL6405.Contig1_All	0.01	4.35	8.76	Spinacia oleracea
CL279.Contig2_All	0.01	2.34	7.87	Fragaria x ananassa
CL3665.Contig1_All	0.01	0.2	4.32	Spinacia oleracea
CL5563.Contig2_All	0.01	0.53	5.73	Citrus sinensis
Unigene31890_All	0.01	0.97	6.60	Morus notabilis
Unigene6725_All	0.01	0.17	4.09	Beta vulgaris
Unigene6747_All	0.01	0.07	2.81	Stylosanthes humilis
CL6456.Contig1_All	2.72	5.85	1.10	Eucalyptus grandis
Unigene17794_All	0.24	1.63	2.76	Spinacia oleracea
Unigene15162_All	0.57	2	1.81	Nelumbo nucifera
CL9481.Contig2_All	0.3	0.76	1.34	Spinacia oleracea
Unigene27958_All	1.62	3.79	1.23	Phoenix dactylifera
CL9189.Contig2_All	0.16	0.08	-1.00	Beta vulgaris
Unigene3282_All	0.54	0.18	-1.58	Spinacia oleracea
Unigene5115_All	1.5	0.5	-1.58	Vitis vinifera
Unigene14070_All	0.08	0.01	-3.00	Spinacia oleracea
Unigene3299_All	0.08	0.01	-3.00	Spinacia oleracea
Unigene4101_All	0.19	0.01	-4.25	Beta vulgaris
CL3665.Contig5_All	3.04	1.2	-1.34	Spinacia oleracea
CL4649.Contig2_All	4.18	0.59	-2.82	Vitis vinifera
CL6405.Contig2_All	8.57	3.66	-1.23	Spinacia oleracea
**GLP**				
CL3077.Contig3_All	0.12	0.01	-3.58	Prunus mume
CL5206.Contig1_All	0.65	0.11	-2.56	Glycine max
Unigene15617_All	1.36	0.01	-7.09	Solanum lycopersicum
Unigene1821_All	3.81	1.19	-1.68	Nelumbo nucifera
CL5206.Contig2_All	0.3	0.91	1.60	Eucalyptus grandis
CL7667.Contig2_All	0.45	0.93	1.05	Vitis vinifera

Unigene26176_All	0.48	3.88	3.01	Nelumbo nucifera
Unigene7248_All	3.28	6.92	1.08	Solanum tuberosum
CL2517.Contig3_All	1.11	3.67	1.73	Chenopodium quinoa
CL585.Contig2_All	0.78	1.56	1.00	Vitis vinifera
CL2975.Contig1_All	1.09	2.35	1.11	Vitis vinifera
Unigene3088_All	0.01	0.42	5.39	Atriplex lentiformis
Unigene5013_All	0.01	0.22	4.46	Mesembryanthemum crystallinum
**CAT**				
CL9179.Contig1_All	1.24	0.01	-6.954	Suaeda salsa
**PEX**				
CL1753.Contig5_All	3.85	17.05	2.15	Eucalyptus grandis
CL8785.Contig7_All	0.01	0.48	5.58	Solanum lycopersicum
CL395.Contig12_All	2.63	1.27	-1.05	Medicago truncatula
**Trx**				
CL4502.Contig2_All	3.34	0.01	-8.38	Morus notabilis
Unigene4304_All	2.01	0.01	-7.65	Nicotiana tabacum
Unigene36570_All	1.14	0.01	-6.83	Theobroma cacao
CL1197.Contig2_All	0.96	0.01	-6.58	Nicotiana sylvestris
CL630.Contig8_All	2.95	0.98	-1.59	Populus trichocarpa
Unigene5866_All	0.04	0.24	2.58	Vitis vinifera
CL5985.Contig2_All	0.53	2.67	2.33	Theobroma cacao
Unigene1940_All	0.13	0.26	1.00	Prunus mume
**SOD**				
CL1360.Contig2_All	53.61	16.21	-1.73	Chenopodiastrum murale
CL2699.Contig1_All	42.13	18.35	-1.20	pinacia oleracea
CL2699.Contig2_All	92.71	17.12	-2.44	pinacia oleracea
CL5754.Contig2_All	1.54	0.01	-7.27	Caragana jubata
CL9769.Contig1_All	4.15	1.69	-1.30	Caragana jubata
CL5119.Contig3_All	2.31	6.18	1.42	Nicotiana tomentosiformis

Table S11. Differentially expressed genes (DEGs) related to enzymatic antioxidant defense systems in roots of *A. canescens* after 100 mM NaCl treatment for 6 h. FPKM-CR6 and FPKM-SR6 respectively indicates the FPKM value of a gene in roots under control condition for 6 h and salt treatment for 6 h. Fold change equals to log2 (FPKM-SR6 / FPKM-CR6). Protein refer to the protein encoded by each DEGs.

Gene ID	FPKM-CR6	FPKM-SR6	Fold change	*Homologous species*
**Grx**				
Unigene2548_All	1.4	127.23	6.51	*Medicago truncatula*
Unigene13286_All	2.77	196.3	6.15	*Malus domestica*
Unigene10362_All	10.72	80.63	2.91	*Prunus mume*

Unigene1514_All	9.95	39.91	2.00	*Prunus mume*
Unigene8875_All	6.2	1.2	-2.37	*Vitis vinifera*
CL6408.Contig1_All	16.39	6.41	-1.35	*Vitis vinifera*
**APX**				
CL2142.Contig5_All	0.63	3.07	2.28	*Glycine max*
Unigene14065_All	66.42	195.12	1.55	*Beta vulgaris*
CL546.Contig2_All	18.36	43.04	1.23	*Spinacia oleracea*
CL6261.Contig2_All	1535.42	33.99	-5.50	*Mesembryanthemum crystallinum*
Unigene10495_All	407.65	67.89	-2.59	*Mesembryanthemum crystallinum*
Unigene6976_All	373.03	135.88	-1.46	*Spinacia oleracea*
**MDAR**				
Unigene3624_All	17.43	51.72	1.57	*Populus trichocarpa*
CL5271.Contig2_All	3.33	0.01	-8.38	*Glycine max*
**DHAR**				
Unigene3279_All	9.54	65.18	2.77	*Spinacia oleracea*
**GR**				
CL6002.Contig1_All	7.86	31.31	1.99	*Dimocarpus longan*
**GST**				
CL7491.Contig4_All	0.57	55.53	6.61	*Salicornia brachiata*
CL7491.Contig3_All	10.17	26.87	1.40	*Salicornia brachiata*
Unigene1347_All	0.52	45.29	6.44	*Vitis vinifera*
Unigene13945_All	12.03	24.8	1.04	*Capsicum chinense*
CL1382.Contig4_All	11.32	0.01	-10.14	*Dianthus caryophyllus*
Unigene16620_All	118.3	0.01	-13.53	*Eucalyptus grandis*
Unigene13885_All	19.39	0.01	-10.92	*Malus domestica*
Unigene29351_All	87.79	0.1	-9.78	*Eucalyptus grandis*
CL1984.Contig1_All	6.87	0.01	-9.42	*Fragaria vesca subsp. vesca*
Unigene12753_All	5.32	0.01	-9.06	*Nelumbo nucifera*
CL2520.Contig2_All	3.57	0.01	-8.48	*Vitis vinifera*
CL9647.Contig1_All	629.6	113.16	-2.48	*Dianthus caryophyllus*
Unigene8569_All	19.91	1.28	-3.96	*Cicer arietinum*
Unigene30938_All	40.81	2.83	-3.85	*Fragaria vesca subsp. vesca*
Unigene18323_All	16.2	2.25	-2.85	*Nelumbo nucifera*
Unigene7483_All	4.6	1.12	-2.04	*Salicornia brachiata*
**GSH-Px**				
CL4674.Contig3_All	0.01	5.07	8.99	*Theobroma cacao*
CL9800.Contig4_All	0.01	2.83	8.14	*Spinacia oleracea*
Unigene1484_All	8.51	34.78	2.03	*Vitis vinifera*
CL4674.Contig2_All	3.01	0.01	-8.23	
CL8077.Contig2_All	12.95	2.96	-2.13	*Theobroma cacao*
Unigene4949_All	21	9.89	-1.09	*Nicotiana sylvestris*
**POD**				

CL6405.Contig2_All	0.59	7.29	3.63	*Spinacia oleracea*
CL6553.Contig2_All	0.6	59.55	6.63	*Eucalyptus grandis*
Unigene12176_All	1.66	113.26	6.09	*Spinacia oleracea*
CL8506.Contig2_All	6.96	14.15	1.02	*Spinacia oleracea*
Unigene1571_All	5.93	24.94	2.07	*Spinacia oleracea*
Unigene5807_All	11.62	35.85	1.63	*Nelumbo nucifera*
CL1702.Contig1_All	109.26	0.01	-13.42	*Spinacia oleracea*
CL1901.Contig1_All	79.81	3.32	-4.59	*Nelumbo nucifera*
CL1981.Contig1_All	29.04	13.47	-1.11	*Spinacia oleracea*
CL2372.Contig2_All	10.01	0.01	-9.97	*Citrus sinensis*
CL279.Contig1_All	34.16	3.11	-3.46	*Fragaria x ananassa*
CL3217.Contig2_All	42.49	0.88	-5.59	*Eucalyptus grandis*
CL3665.Contig1_All	303.91	0.01	-14.89	*Spinacia oleracea*
CL4099.Contig1_All	91.92	45.65	-1.01	*Camellia oleifera*
CL4649.Contig1_All	3.01	0.01	-8.23	*Vitis vinifera*
CL5966.Contig1_All	364.97	0.26	-10.46	*Nelumbo nucifera*
CL6456.Contig4_All	124.14	16.87	-2.88	*Phoenix dactylifera*
CL7743.Contig1_All	12.27	0.14	-6.45	*Spinacia oleracea*
CL7926.Contig1_All	18.5	0.48	-5.27	*Prunus mume*
CL8547.Contig1_All	4.31	0.01	-8.75	*Malus domestica*
CL9189.Contig2_All	9.87	0.01	-9.95	*Beta vulgaris*
CL9560.Contig1_All	151.39	74.64	-1.02	*Phoenix dactylifera*
Unigene12967_All	2.41	0.01	-7.91	*Malus domestica*
Unigene13832_All	24.27	0.01	-11.24	*Theobroma cacao*
Unigene14000_All	647.44	18.81	-5.11	*Spinacia oleracea*
Unigene14070_All	251	0.16	-10.62	*Spinacia oleracea*
Unigene14074_All	42.01	13.91	-1.59	*Theobroma cacao*
Unigene15162_All	99.41	10.59	-3.23	*Nelumbo nucifera*
Unigene17531_All	123.64	4.01	-4.95	*Sesuvium portulacastrum*
Unigene17794_All	104.12	0.49	-7.73	*Spinacia oleracea*
Unigene1784_All	16.62	1.21	-3.78	*Citrus sinensis*
Unigene21270_All	881.6	40.77	-4.43	*Nelumbo nucifera*
Unigene229_All	3.17	0.01	-8.31	*Pyrus x bretschneideri*
Unigene27160_All	20.48	1.34	-3.93	*Nelumbo nucifera*
Unigene27958_All	40.86	7.61	-2.42	*Phoenix dactylifera*
Unigene3282_All	137.28	0.01	-13.74	*Spinacia oleracea*
Unigene3299_All	13.34	0.17	-6.29	*Spinacia oleracea*
Unigene4101_All	7.61	0.01	-9.57	*Beta vulgaris*
Unigene5115_All	52.03	0.84	-5.95	*Vitis vinifera*
Unigene5170_All	73.03	0.78	-6.55	*Nicotiana tabacum*
Unigene6725_All	69.54	0.01	-12.76	*Beta vulgaris*
Unigene6747_All	142.08	0.01	-13.79	*Stylosanthes humilis*
Unigene6922_All	33.1	0.01	-11.69	*Pyrus x bretschneideri*
Unigene8100_All	1.82	0.01	-7.51	*Musa acuminata subsp. malaccensis*

Unigene8726_All	216.66	0.69	-8.29	*Vitis vinifera*
Unigene8799_All	16.1	0.01	-10.65	
Unigene9514_All	3.72	0.01	-8.54	*Fragaria vesca subsp. vesca*
**GLP**				
CL2517.Contig3_All	3.73	0.8	-2.22	*Glycine max*
CL2975.Contig1_All	10.63	1.43	-2.89	*Vitis vinifera*
CL3077.Contig3_All	44.94	0.01	-12.13	*Prunus mume*
CL4866.Contig2_All	23.86	0.01	-11.22	*Beta vulgaris*
CL5206.Contig1_All	1266.39	0.32	-11.95	*Glycine max*
CL585.Contig1_All	8.06	0.01	-9.65	*Vitis vinifera*
CL7262.Contig1_All	3.12	0.01	-8.29	*Beta vulgaris*
CL7667.Contig1_All	80.29	0.38	-7.72	*Atriplex lentiformis*
CL7904.Contig1_All	156.97	0.01	-13.94	*Beta vulgaris*
Unigene12529_All	49.27	0.01	-12.27	*Beta vulgaris*
Unigene12866_All	81.41	3.92	-4.38	*Atriplex lentiformis*
Unigene13948_All	262.08	0.2	-10.36	*Atriplex lentiformis*
Unigene16326_All	25.61	1.52	-4.07	*Beta vulgaris*
Unigene1703_All	63.69	0.01	-12.64	*Prunus mume*
Unigene1714_All	19.89	9.45	-1.07	*Fragaria vesca subsp. vesca*
Unigene3088_All	1218.1	0.64	-10.89	*Atriplex lentiformis*
Unigene3091_All	137.58	23.76	-2.53	*Vitis vinifera*
Unigene3132_All	603.98	1.04	-9.18	*Nelumbo nucifera*
Unigene5013_All	292.63	0.11	-11.38	*Mesembryanthemum crystallinum*
Unigene7967_All	7.27	0.01	-9.51	*Beta vulgaris*
Unigene7248_All	0.01	8.16	9.67	*Solanum tuberosum*
**CAT**				
CL9309.Contig1_All	3.03	12.95	2.10	*Suaeda salsa*
CL9179.Contig2_All	114.47	457.86	1.20	*Suaeda salsa*
**PEX**				
CL395.Contig12_All	3.4	0.01	-8.41	*Medicago truncatula*
CL638.Contig6_All	13.62	1.64	-3.05	*Medicago truncatula*
CL8785.Contig4_All	3.34	0.01	-8.38	*Theobroma cacao*
Unigene9291_All	13.28	5.95	-1.16	*Solanum lycopersicum*
Unigene7436_All	28.23	4.38	-2.69	*Nelumbo nucifera*
CL9371.Contig1_All	20.86	8.58	-1.28	*Cucumis melo*
Unigene3397_All	10.47	4.96	-1.08	*Fragaria vesca subsp. vesca*
Unigene14919_All	68.34	33.51	-1.03	*Nelumbo nucifera*
CL8126.Contig2_All	46.72	20.6	-1.18	*Vitis vinifera*
Unigene16102_All	0.22	11.54	5.71	*Glycine max*
CL2499.Contig4_All	26.46	628.16	4.57	*Spinacia oleracea*
Unigene28652_All	0.84	15.66	4.22	*Vitis vinifera*
CL2499.Contig7_All	36.21	625.93	4.11	*Spinacia oleracea*
Unigene18788_All	0.25	3.43	3.78	*Vitis vinifera*

CL2602.Contig1_All	1.17	11.64	3.31	*Vitis vinifera*
CL4210.Contig2_All	0.58	3.6	2.63	*Vitis vinifera*
CL3399.Contig3_All	2.97	12.76	2.10	*Nicotiana tomentosiformis*
CL5507.Contig2_All	22.94	71.41	1.64	*Vitis vinifera*
Unigene2492_All	17.5	54.39	1.64	*Theobroma cacao*
CL3148.Contig2_All	2.96	7.83	1.40	*Solanum lycopersicum*
CL5507.Contig3_All	31.12	78.59	1.34	*Vitis vinifera*
Unigene8760_All	7.39	16.83	1.19	*Medicago truncatula*
**Trx**				
CL2824.Contig1_All	1.32	238.95	7.50	*Spinacia oleracea*
CL4320.Contig1_All	10.15	81.07	3.00	*Vitis vinifera*
CL5614.Contig1_All	0.99	43	5.44	*Brassica rapa*
CL630.Contig7_All	13.73	30.36	1.14	*Solanum tuberosum*
Unigene6798_All	0.65	515.84	9.63	*Spinacia oleracea*
Unigene13861_All	4.77	57.66	3.60	*Eucalyptus grandis*
Unigene6795_All	9.81	63.79	2.70	*Cucumis melo*
Unigene8536_All	1.03	6.4	2.64	*Theobroma cacao*
Unigene5401_All	7.07	40.14	2.51	*Eucalyptus grandis*
CL8815.Contig2_All	3.94	21.32	2.44	*Malus domestica*
Unigene5160_All	15.74	43.22	1.46	*Theobroma cacao*
Unigene12286_All	14.62	38.85	1.41	*Theobroma cacao*
Unigene10424_All	13.99	30.23	1.11	*Prunus mume*
Unigene12087_All	2.43	453.48	7.54	*Spinacia oleracea*
Unigene3533_All	14.36	115.19	3.00	*Spinacia oleracea*
CL7936.Contig1_All	3.38	145.43	5.43	*Suaeda salsa*
Unigene18720_All	1940.74	764.7	-1.34	
Unigene12094_All	48.24	13.28	-1.86	*Tamarix hispida*
Unigene12308_All	44.42	12.01	-1.89	*Fragaria vesca subsp. vesca*
Unigene1940_All	6.68	0.56	-3.58	*Prunus mume*
Unigene5866_All	2.34	0.12	-4.29	*Vitis vinifera*
CL1197.Contig1_All	2.28	0.01	-7.83	*Nicotiana sylvestris*
Unigene5027_All	48.74	12.4	-1.97	*Cucumis melo*
**PrxR**				
Unigene13818_All	3.81	38.69	3.34	*Vitis vinifera*
Unigene5142_All	30.34	198.95	2.71	*Tamarix hispida*
CL7966.Contig2_All	7.35	37.87	2.37	*Cicer arietinum*
Unigene1465_All	47.6	95.22	1.00	*Tamarix hispida*
CL7936.Contig1_All	3.38	145.43	5.43	*Suaeda salsa*
**SOD**				
CL5119.Contig3_All	0.01	8.8	9.78	*Suaeda salsa*
Unigene5793_All	45.92	353.49	2.94	*Phytolacca acinosa*
CL1360.Contig2_All	12.23	73.9	2.60	*Chenopodiastrum murale*
CL2699.Contig2_All	9.67	112.87	3.55	*Suaeda salsa*
Unigene3542_All	13.19	29.04	1.14	*Tamarix hispida*

Unigene35175_All	1.87	0.01	-7.55	*Vitis vinifera*

Table S12. Differentially expressed genes (DEGs) related to enzymatic antioxidant defense systems in roots of *A. canescens* after 100 mM NaCl treatment for 24 h. FPKM-CR24 and FPKM-SR24 respectively indicates the FPKM value of a gene in roots under control condition for 24 h and salt treatment for 24 h. Fold change equals to log2 (FPKM-SR24 / FPKM-CR24). Protein refer to the protein encoded by each DEGs.

Gene ID	FPKM-CR24	FPKM-SR24	Fold change	Homologous species
**GLR**				
CL3957.Contig2_All	0.01	1.04	6.70	*Rheum australe*
Unigene40963_All	0.66	1.35	1.03	*Coccomyxa subellipsoidea*
Unigene2548_All	0.85	10.15	3.58	*Medicago truncatula*
Unigene39972_All	0.57	0.01	-5.83	*Ricinus communis*
Unigene16019_All	4.01	1.44	-1.48	*Rheum australe*
**APX**				
CL2860.Contig2_All	0.01	1.58	7.30	*Glycine max*
Unigene23148_All	0.01	0.85	6.41	*Theobroma cacao*
CL2142.Contig1_All	0.01	0.34	5.09	*Vitis vinifera*
**MDAR**				
CL5271.Contig1_All	1.09	2.78	1.35	*Glycine max*
**GST**				
CL7491.Contig3_All	0.01	4.55	8.83	*Salicornia brachiata*
Unigene39828_All	0.01	0.81	6.34	*Malva pusilla*
CL7491.Contig1_All	0.39	1.01	1.37	*Salicornia brachiata*
Unigene20291_All	0.29	0.6	1.05	*Knorringia sibirica*
Unigene25569_All	0.17	0.34	1.00	*Theobroma cacao*
CL2520.Contig2_All	12.86	5.09	-1.34	*Vitis vinifera*
Unigene20044_All	6.52	2.49	-1.39	*Solanum lycopersicum*
Unigene18893_All	0.56	0.23	-1.28	*Brassica rapa*
**GSH-Px**				
CL9800.Contig1_All	0.01	0.96	6.58	*Spinacia oleracea*
CL8077.Contig1_All	2.2	0.01	-7.78	*Theobroma cacao*
CL9800.Contig4_All	2.09	0.37	-2.50	*Spinacia oleracea*
CL4674.Contig3_All	5	1.12	-2.16	*Theobroma cacao*
**POD**				
CL5563.Contig2_All	1.28	2.86	1.16	*Citrus sinensis*
CL6456.Contig3_All	5.34	12.25	1.20	*Eucalyptus grandis*
CL7926.Contig3_All	18.71	44.18	1.24	*Ricinus communis*
Unigene35178_All	0.61	2.5	2.04	*Prunus mume*
CL4649.Contig1_All	4.5	1.2	-1.91	*Vitis vinifera*
CL8547.Contig1_All	11.67	5.54	-1.07	*Malus domestica*
Unigene12755_All	15.87	4.73	-1.75	*Vitis vinifera*
Unigene12967_All	10.87	2.86	-1.93	*Malus domestica*

Unigene1784_All	77.31	22.04	-1.81	*Citrus sinensis*
Unigene21864_All	0.53	0.01	-5.73	*Fragaria vesca*
Unigene34540_All	0.76	0.01	-6.25	*Fragaria vesca*
Unigene5807_All	3.28	1.12	-1.55	*Nelumbo nucifera*
Unigene8100_All	7.66	1.78	-2.11	*Musa acuminata*
**GLP**				
CL2517.Contig3_All	2.24	4.92	1.14	*Glycine max*
Unigene12866_All	72.15	174.43	1.27	*Atriplex lentiformis*
Unigene36054_All	0.54	3.89	2.85	*Vitis vinifera*
CL7262.Contig1_All	6.12	13.64	1.16	*Beta vulgaris*
CL3077.Contig2_All	1.35	0.01	-7.08	*Prunus mume*
CL5206.Contig2_All	0.95	0.33	-1.53	*Eucalyptus grandis*
CL7262.Contig2_All	5.09	2.54	-1.00	*Beta vulgaris*
Unigene15617_All	7.7	1.76	-2.13	*Solanum lycopersicum*
Unigene29127_All	2.23	0.98	-1.19	*Theobroma cacao*
**CAT**				
Unigene30829_All	0.91	0.01	-6.51	*Phytophthora infestans*
**PEX**				
CL8785.Contig7_All	0.01	3.83	8.58	*Solanum lycopersicum*
CL395.Contig12_All	0.01	2.12	7.73	*Medicago truncatula*
CL8785.Contig5_All	7.07	0.6	-3.56	*Pyrus x bretschneideri*
CL1753.Contig2_All	3.71	0.01	-8.54	*Eucalyptus grandis*
**Trx**				
Unigene36570_All	0.01	0.41	5.36	*Theobroma cacao*
CL3467.Contig1_All	3.88	9.13	1.23	*Vitis vinifera*
Unigene6798_All	0.33	1.47	2.16	*Spinacia oleracea*
CL2824.Contig1_All	1.33	3.3	1.31	*Spinacia oleracea*
Unigene25602_All	0.55	1.12	1.03	*Fragaria vesca*
Unigene20397_All	1.94	0.93	-1.06	*Citrus sinensis*
**SOD**				
CL5119.Contig2_All	0.01	0.55	5.78	*Spinacia oleracea*
CL5754.Contig2_All	0.79	0.01	-6.30	*Caragana jubata*
Unigene39937_All	0.61	0.01	-5.93	*Ostreococcus tauri*

Table S13. Differentially expressed genes (DEGs) related to transcription factor in leaves of *A. canescens* after 100 mM NaCl treatment for 6 h. FPKM-CL6 and FPKM-SL6 respectively indicates the FPKM value of a gene in leaves under control condition for 6 h and salt treatment for 6 h. Fold change equals to log2 (FPKM-SL6 / FPKM-CL6). Protein refer to the protein encoded by each DEGs.

Gene ID	FPKM -CL6	FPKM -SL6	Fold change	*Homologous species*
**WRKY**				
CL1071.Contig2_All	95.08	0.01	-13.21	*Spinacia oleracea*
Unigene3786_All	5.24	2.34	-1.16	*Vitis vinifera*

Unigene1946_All	4.76	10.67	1.16	*Tamarix hispida*
Unigene6298_All	15.55	42.37	1.45	*Pyrus x bretschneideri*
CL380.Contig2_All	3.75	12.16	1.70	*Nelumbo nucifera*
CL7772.Contig1_All	1.56	5.62	1.85	*Ricinus communis*
Unigene5485_All	3.49	15.61	2.16	*Populus tomentosa*
CL3186.Contig1_All	3.97	18.5	2.22	*Tamarix hispida*
CL3276.Contig2_All	0.76	5.11	2.75	*Theobroma cacao*
Unigene17677_All	1.37	9.5	2.79	*Panax quinquefolius*
CL7561.Contig1_All	3.1	23.45	2.92	*Theobroma cacao*
Unigene26885_All	2.35	24.64	3.39	*Tamarix hispida*
Unigene12295_All	2.18	23.35	3.42	*Vitis vinifera*
CL4275.Contig2_All	2.36	48.53	4.36	*Vitis vinifera*
Unigene11100_All	0.26	8.04	4.95	*Nicotiana tomentosiformis*
Unigene9476_All	0.14	10.48	6.23	*Vitis vinifera*
CL629.Contig1_All	0.45	122.36	8.09	*Vitis vinifera*
Unigene1538_All	0.01	1.91	7.58	*Solanum tuberosum*
Unigene40205_All	0.01	3.74	8.55	*Theobroma cacao*
Unigene12958_All	0.01	8.68	9.76	*Citrus sinensis*
Unigene5531_All	0.01	14.2	10.47	*Citrus sinensis*
Unigene16243_All	0.01	20.69	11.01	*Theobroma cacao*
**MYB**				
Unigene13756_All	3.01	0.01	-8.23	*Malus domestica*
Unigene21553_All	2.13	0.01	-7.73	*Medicago truncatula*
Unigene3069_All	24.85	0.32	-6.28	*Theobroma cacao*
Unigene12759_All	5.29	0.33	-4.00	*Jatropha curcas*
Unigene4919_All	3.41	0.54	-2.66	*Fragaria vesca*
CL422.Contig3_All	8.21	1.45	-2.50	*Nelumbo nucifera*
Unigene7861_All	13.66	3.84	-1.83	*Arachis hypogaea*
Unigene7260_All	32.16	70.13	1.12	*Tamarix hispida*
CL5601.Contig1_All	6.15	14.17	1.20	*Morus notabilis*
Unigene11056_All	28.1	102.02	1.86	*Salicornia brachiata*
Unigene9243_All	2.28	9.8	2.10	*Theobroma cacao*
CL2987.Contig1_All	1.3	6.92	2.41	*Ricinus communis*
CL6822.Contig3_All	4.21	23.47	2.48	*Vitis vinifera*
Unigene15334_All	0.35	2.04	2.54	*Nicotiana tomentosiformis*
CL4970.Contig1_All	38.77	229.8	2.57	*Nicotiana tabacum*
Unigene15802_All	1.04	7.75	2.90	*Nicotiana sylvestris*
Unigene12857_All	3.76	37.73	3.33	*Populus trichocarpa*
CL5476.Contig1_All	0.32	3.43	3.42	*Jatropha curcas*
Unigene7714_All	0.63	11	4.13	*Glycine max*
CL5951.Contig4_All	4.88	88.94	4.19	*Theobroma cacao*
CL7820.Contig3_All	0.26	5.73	4.46	*Nelumbo nucifera*
Unigene17573_All	0.53	17.09	5.01	*Jatropha curcas*
Unigene10405_All	0.16	17.86	6.80	*Theobroma cacao*

Unigene10098_All	0.01	2.12	7.73	*Medicago truncatula*
Unigene35460_All	0.01	2.3	7.85	*Prunus mume*
Unigene28181_All	0.01	3.65	8.51	*Theobroma cacao*
Unigene403_All	0.01	8.4	9.71	*Theobroma cacao*
CL8721.Contig1_All	0.01	9.9	9.95	*Populus trichocarpa*
CL1223.Contig1_All	0.01	45.2	12.14	*Theobroma cacao*
CL4043.Contig2_All	0.01	56.27	12.46	*Arachis hypogaea*
**HD-ZIP/bZIP**				
CL1844.Contig2_All	0.58	26.42	5.51	*Populus trichocarpa*
CL1962.Contig1_All	8.48	17.27	1.03	*Vitis vinifera*
CL5048.Contig3_All	4.33	32.82	2.92	*Spinacia oleracea*
CL6157.Contig1_All	0.29	4.48	3.95	*Vitis vinifera*
Unigene11050_All	0.39	0.97	1.31	*Nicotiana tomentosiformis*
Unigene12290_All	9.81	55.3	2.49	*Theobroma cacao*
Unigene15839_All	0.83	19.5	4.55	*Prunus mume*
Unigene15896_All	28.68	98.37	1.78	*Nicotiana tomentosiformis*
Unigene1814_All	44.08	111.52	1.34	*Tamarix hispida*
Unigene534_All	6.23	13.61	1.13	*Theobroma cacao*
Unigene5526_All	1.32	3.24	1.30	*Solanum lycopersicum*
Unigene5448_All	3.09	8.06	1.38	*Vitis vinifera*
Unigene4320_All	12.27	36.64	1.58	*Vitis vinifera*
Unigene9492_All	0.29	1.01	1.80	*Theobroma cacao*
Unigene221_All	1.41	6.67	2.24	*Tamarix hispida*
Unigene5773_All	6.03	38.55	2.68	*Tamarix hispida*
Unigene2090_All	22.22	1.98	-3.49	*Solanum tuberosum*
Unigene26257_All	2.31	0.01	-7.85	*Nelumbo nucifera*
Unigene26259_All	1.22	0.01	-6.93	*Prunus mume*
Unigene23519_All	1.05	0.01	-6.71	*Vitis vinifera*
Unigene23520_All	0.55	0.01	-5.78	*Nicotiana sylvestris*
Unigene7774_All	7.01	0.21	-5.06	*Vitis vinifera*
Unigene36315_All	0.01	0.46	5.52	*Malus domestica*
Unigene27471_All	0.01	1.22	6.93	*Nicotiana sylvestris*
Unigene16296_All	0.01	5.43	9.08	*Vitis vinifera*
Unigene14640_All	0.01	13.94	10.45	*Theobroma cacao*
**AP2/ERF**				
CL3234.Contig1_All	9.72	1.12	-3.12	*Rumex acetosa*
Unigene18547_All	1.07	13.93	3.70	*Theobroma cacao*
CL5582.Contig1_All	1.75	13.19	2.91	*Citrus sinensis*
CL5582.Contig2_All	0.51	5.51	3.43	*Pyrus x bretschneideri*
CL4440.Contig1_All	0.85	9.07	3.42	*Eucalyptus grandis*
Unigene18548_All	0.63	6.13	3.28	*Theobroma cacao*
Unigene27753_All	0.75	4.81	2.68	*Nicotiana sylvestris*
Unigene31276_All	0.94	5.73	2.61	*Pyrus x bretschneideri*
Unigene14637_All	108.37	418.32	1.95	*Solanum lycopersicum*

Unigene14464_All	4.43	12.89	1.54	*Theobroma cacao*
Unigene12257_All	2.21	5.22	1.24	*Nelumbo nucifera*
Unigene7169_All	0.27	13.5	5.64	*Solanum tuberosum*
Unigene3600_All	0.25	11.35	5.50	*Theobroma cacao*
CL7480.Contig2_All	1.69	26.86	3.99	*Pyrus x bretschneideri*
Unigene17627_All	3.23	17.08	2.40	*Nelumbo nucifera*
Unigene17680_All	142.45	450.85	1.66	*Vitis vinifera*
Unigene5762_All	7.42	23.05	1.64	*Morus notabilis*
Unigene6756_All	28.34	60.26	1.09	*Populus trichocarpa*
Unigene6706_All	2.15	25.46	3.57	*Tamarix hispida*
CL3234.Contig2_All	20.28	44.21	1.12	*Rumex acetosa*
Unigene1741_All	0.01	1.89	7.56	*Malus domestica*
CL1411.Contig1_All	0.01	15.38	10.59	*Morus notabilis*
Unigene3750_All	0.01	8.93	9.80	*Theobroma cacao*
**DREB**				
Unigene3588_All	18.94	77.31	2.03	*Salicornia brachiata*
Unigene15818_All	3.99	99.39	4.64	*Suaeda salsa*
CL5907.Contig1_All	10.64	30.24	1.51	*Salicornia brachiata*
**NAC**				
Unigene167_All	0.01	13.85	10.44	*Theobroma cacao*
Unigene10236_All	0.2	5.75	4.85	*Tamarix hispida*
Unigene12962_All	0.37	7.81	4.40	*Pyrus x bretschneideri*
CL9208.Contig1_All	5.63	68.23	3.60	*Morus notabilis*
Unigene12773_All	0.87	8.2	3.24	*Hevea brasiliensis*
CL2948.Contig2_All	5.31	29.79	2.49	*Theobroma cacao*
Unigene31695_All	0.38	3.15	3.05	*Vitis vinifera*
Unigene1980_All	1.36	8.27	2.60	*Ricinus communis*
Unigene3303_All	2.55	14.93	2.55	*Theobroma cacao*
Unigene16456_All	1.83	10.15	2.47	*Suaeda liaotungensis*
Unigene18478_All	0.62	3.17	2.35	*Theobroma cacao*
Unigene6031_All	1.51	7.55	2.32	*Nelumbo nucifera*
Unigene1286_All	2.18	10.81	2.31	*Fragaria vesca subsp. Vesca*
Unigene3569_All	4.1	19.92	2.28	*Theobroma cacao*
Unigene6877_All	0.91	4.31	2.24	*Tamarix hispida*
CL6151.Contig2_All	2.7	10.97	2.02	*Pyrus x bretschneideri*
Unigene4165_All	57.15	180.53	1.66	*Ricinus communis*
CL8594.Contig3_All	49.1	134.24	1.45	*Suaeda liaotungensis*
CL9299.Contig2_All	6.58	16.85	1.36	*Jatropha curcas*
Unigene15768_All	48.34	119.49	1.31	*Suaeda liaotungensis*
Unigene16846_All	56.9	127.81	1.17	*Jatropha curcas*
Unigene17664_All	12.64	28.13	1.15	*Phaseolus vulgaris*
CL3.Contig1_All	2.13	4.55	1.10	*Theobroma cacao*
Unigene17764_All	9.73	0.01	-9.93	*Gossypium hirsutum*
Unigene8630_All	8	3.02	-1.41	*Pyrus x bretschneideri*

**bHLH**				
Unigene1766_All	0.01	29.31	11.52	*Nicotiana sylvestris*
Unigene16064_All	0.01	25.27	11.30	*Prunus mume*
Unigene7013_All	0.01	3.01	8.23	*Vitis vinifera*
CL9130.Contig1_All	0.01	1.89	7.56	*Citrus sinensis*
Unigene27074_All	0.01	1.08	6.75	*Fragaria vesca*
Unigene10520_All	0.01	16.83	10.72	*Lotus japonicus*
Unigene4053_All	0.01	2.65	8.05	*Populus simonii*
Unigene16138_All	0.38	20.66	5.76	*Nelumbo nucifera*
Unigene17695_All	2.64	81.65	4.95	*Vitis vinifera*
Unigene12084_All	3.38	57.08	4.08	*Malus domestica*
Unigene11162_All	0.27	2.94	3.44	*Solanum lycopersicum*
Unigene7417_All	0.46	2.67	2.54	*Vitis vinifera*
CL9504.Contig1_All	3.43	16.48	2.26	*Vitis vinifera*
Unigene17775_All	3.18	15.14	2.25	*Malus domestica*
Unigene17676_All	1.33	6.14	2.21	*Fragaria vesca*
Unigene38100_All	5.12	13.72	1.42	*Nelumbo nucifera*
Unigene14971_All	29.74	74.03	1.32	*Vitis vinifera*
Unigene3_All	2.28	5.28	1.21	*Cicer arietinum*
Unigene11068_All	6.78	23.81	1.81	*Theobroma cacao*
Unigene3415_All	8.12	17.96	1.15	*Vitis vinifera*
CL6102.Contig2_All	15.59	33.44	1.10	*Vitis vinifera*
CL7924.Contig1_All	5.84	0.01	-9.19	*Vitis vinifera*
Unigene8694_All	49.65	0.49	-6.66	*Vitis vinifera*
CL3710.Contig1_All	2.67	0.42	-2.67	*Prunus mume*
CL7707.Contig2_All	4.07	0.34	-3.58	*Theobroma cacao*
Unigene27729_All	34.75	17.37	-1.00	*Theobroma cacao*
**MADS-box**				
Unigene24561_All	0.01	10.08	9.98	*Vitis vinifera*
Unigene9423_All	0.01	7.42	9.54	*Nicotiana tomentosiformis*
CL5411.Contig5_All	0.01	19.73	10.95	*Phoenix dactylifera*
CL6533.Contig2_All	0.18	8.45	5.55	*Prunus mume*
CL2234.Contig4_All	0.69	16.8	4.61	*Gossypium hirsutum*
CL1445.Contig5_All	1.98	7.67	1.95	*Beta vulgaris*
CL1478.Contig1_All	4.55	0.01	-8.83	*Nelumbo nucifera*
CL7398.Contig3_All	0.01	40.5	11.98	*Theobroma cacao*
Unigene8363_All	0.1	2.82	4.82	*Theobroma cacao*
CL7870.Contig2_All	1.14	5.91	2.37	*Eucalyptus grandis*
CL6093.Contig1_All	3.42	9.89	1.53	*Theobroma cacao*
CL3061.Contig2_All	4.47	156.33	5.13	*Theobroma cacao*
CL1153.Contig2_All	10.83	23.78	1.13	*Vitis vinifera*
CL1623.Contig2_All	8.52	20.9	1.29	*Pyrus x bretschneideri*
CL1816.Contig1_All	3.71	10.79	1.54	*Theobroma cacao*
CL213.Contig2_All	0.56	12.78	4.51	*Brassica rapa*

CL2494.Contig2_All	2.62	6.97	1.41	*Populus trichocarpa*
CL2555.Contig8_All	0.97	4.14	2.09	*Nicotiana sylvestris*
CL2680.Contig2_All	2.34	5.74	1.29	*[Pyrus x bretschneideri*
CL2784.Contig2_All	2.01	8.77	2.13	*Solanum lycopersicum*
CL2904.Contig7_All	7.1	17.12	1.27	*Citrus sinensis*
CL3061.Contig1_All	1.98	46.4	4.55	*Theobroma cacao*
CL3811.Contig4_All	8.97	75.68	3.08	*Nicotiana sylvestris*
CL4249.Contig1_All	21.42	45.72	1.09	*Populus trichocarpa*
CL4800.Contig1_All	4	8.35	1.06	*Theobroma cacao*
CL4998.Contig3_All	3.1	11.4	1.88	*Nicotiana tomentosiformis*
CL5844.Contig1_All	5.29	13.97	1.40	*Theobroma cacao*
CL6239.Contig1_All	6.1	38.75	2.67	*Glycine max*
CL647.Contig3_All	3.28	12.98	1.98	*Nelumbo nucifera*
CL6754.Contig1_All	2.15	4.92	1.19	*Theobroma cacao*
CL6912.Contig1_All	0.44	17.36	5.30	*Citrus sinensis*
CL7175.Contig1_All	9.82	46.73	2.25	*Nelumbo nucifera*
CL7205.Contig2_All	5.78	13.94	1.27	*Beta vulgaris*
CL8054.Contig2_All	5.22	21.17	2.02	*Nicotiana tomentosiformis*
CL8626.Contig2_All	0.01	9.85	9.94	*Nelumbo nucifera*
Unigene10003_All	7.51	17.24	1.20	*Citrus sinensis*
Unigene10303_All	1.37	7.42	2.44	*Theobroma cacao*
Unigene10320_All	2.26	8.11	1.84	*Theobroma cacao*
Unigene10801_All	0.15	4.36	4.86	*Solanum tuberosum*
Unigene11037_All	9.29	19.5	1.07	*Silene latifolia*
Unigene11889_All	0.15	6.86	5.52	*Ricinus communis*
Unigene12085_All	5.43	24.48	2.17	*Ricinus communis*
Unigene1234_All	1.11	33.6	4.92	*Populus trichocarpa*
Unigene18007_All	0.47	24.56	5.71	*Nicotiana sylvestris*
Unigene1480_All	3.49	108.54	4.96	*Eucalyptus grandis*
Unigene6975_All	1.76	50.95	4.86	*Morus notabilis*
Unigene15982_All	2.78	27.46	3.30	*Populus trichocarpa*
Unigene35464_All	0.56	5.3	3.24	*Solanum lycopersicum*
Unigene16195_All	1.17	8.86	2.92	*Pyrus x bretschneideri*
Unigene31278_All	2.47	18.17	2.88	*Glycine max*
Unigene251_All	0.48	3.02	2.65	*Medicago truncatula*
Unigene1451_All	6.66	40.61	2.61	*Vitis vinifera*
Unigene17988_All	1.82	10.65	2.55	*Vitis vinifera*
Unigene461_All	0.49	2.63	2.42	*Nelumbo nucifera*
Unigene12834_All	4.85	25.1	2.37	*Vitis vinifera*
Unigene3903_All	0.9	3.78	2.07	*Nelumbo nucifera*
Unigene17630_All	10.69	36.66	1.78	*Populus trichocarpa*
Unigene2389_All	6.34	16.72	1.40	*Vitis vinifera*
Unigene18146_All	3.99	9.98	1.32	*Vitis vinifera*
Unigene6232_All	9.14	22.84	1.32	*Ricinus communis*

Unigene16891_All	10.97	27.24	1.31	*Prunus mume*
Unigene2542_All	15.99	37.47	1.23	*Ricinus communis*
Unigene8602_All	5	11.54	1.21	*Nelumbo nucifera*
Unigene8802_All	29.46	65.84	1.16	*Vitis vinifera*
Unigene2405_All	4.5	9.95	1.14	*Eucalyptus grandis*
Unigene8557_All	139.78	307.85	1.14	*Vitis vinifera*
Unigene13066_All	29.19	63.32	1.12	*Populus trichocarpa*
Unigene5790_All	3.25	7.03	1.11	*Theobroma cacao*
Unigene7787_All	4.56	9.77	1.10	*Theobroma cacao*
Unigene17840_All	10.01	21.3	1.09	*Morus notabilis*
Unigene12718_All	46.58	95.29	1.03	*Morus notabilis*
Unigene8812_All	47.64	23.78	-1.00	*Nicotiana sylvestris*
Unigene16749_All	30.3	14.52	-1.06	*Theobroma cacao*
CL6005.Contig2_All	5	2.15	-1.22	*Theobroma cacao*
Unigene7406_All	7.88	2.84	-1.47	*Nelumbo nucifera*
Unigene9369_All	6.72	2.24	-1.58	*Vitis vinifera*
Unigene5811_All	11.02	2.8	-1.98	*Prunus mume*
Unigene9484_All	4.11	0.96	-2.10	*Vitis vinifera*
CL7243.Contig2_All	10.64	1.43	-2.90	*Nelumbo nucifera*
CL6715.Contig1_All	7.04	0.9	-2.97	*Brassica rapa*
Unigene12475_All	7.22	0.71	-3.35	*Medicago truncatula*
Unigene18264_All	20.94	1.99	-3.40	*Populus trichocarpa*
Unigene9454_All	3.86	0.01	-8.59	*Amaranthus hypochondriacus*
CL6690.Contig2_All	77.15	0.01	-12.91	*Vitis vinifera*
CL5054.Contig2_All	9.43	2.11	-2.16	*Cicer arietinum*
CL5550.Contig2_All	29.41	6.7	-2.13	*Glycine max*
CL37.Contig3_All	1.76	0.01	-7.46	*Populus trichocarpa*
CL4442.Contig2_All	17.09	3.61	-2.24	*Vitis vinifera*
CL4536.Contig2_All	35.14	0.01	-11.78	*Solanum lycopersicum*
CL469.Contig1_All	6.71	0.01	-9.39	*Vitis vinifera*
CL2069.Contig1_All	21.5	1.01	-4.41	*Nelumbo nucifera*
CL9583.Contig2_All	10.91	0.21	-5.70	*Saruma henryi*
Unigene7539_All	4.14	0.11	-5.23	*Theobroma cacao*
CL5483.Contig3_All	5.98	0.32	-4.22	*Theobroma cacao*
Unigene12010_All	41.16	10.94	-1.91	*Prunus mume*
CL5689.Contig2_All	14.6	4.12	-1.83	*Theobroma cacao*
Unigene7177_All	13.23	4.34	-1.61	*Vitis vinifera*
CL6461.Contig1_All	8.74	3.17	-1.46	*Theobroma cacao*
CL2153.Contig1_All	3.2	0.54	-2.57	*Theobroma cacao*
**HSF**				
CL3372.Contig2_All	0.01	26.48	11.37	*Cucumis melo*
CL7682.Contig2_All	0.01	9.88	9.95	*Vitis vinifera*
CL1745.Contig1_All	5	15.62	1.64	*Populus trichocarpa*

CL1986.Contig1_All	0.3	3.37	3.49	*Capparis spinosa*
CL2066.Contig3_All	4.08	26.94	2.72	*Citrus sinensis*
CL2779.Contig1_All	3.8	7.66	1.01	*Vitis vinifera*
CL3494.Contig2_All	13.4	42.15	1.65	*Oxybasis rubra*
CL4848.Contig2_All	1.95	8.28	2.09	*Theobroma cacao*
CL5651.Contig2_All	7.09	26.27	1.89	*Pyrus x bretschneideri*
CL6191.Contig2_All	1.19	4.23	1.83	*Chenopodium album*
CL6546.Contig6_All	2.65	6.27	1.24	*Theobroma cacao*
CL683.Contig2_All	0.12	1.85	3.95	*Citrus sinensis*
CL7113.Contig1_All	1.44	13.08	3.18	*Cucumis melo*
CL8208.Contig1_All	1.38	13.54	3.29	*Vitis vinifera*
Unigene10400_All	4.86	15.94	1.71	*Medicago truncatula*
Unigene12840_All	0.52	12.14	4.55	*Nicotiana tomentosiformis*
Unigene8347_All	0.16	2.03	3.67	*Vitis vinifera*
Unigene5261_All	2.89	34.42	3.57	*Pyrus x bretschneideri*
Unigene18326_All	3.01	26.35	3.13	*Nicotiana tomentosiformis*
Unigene3969_All	2.42	17.48	2.85	*Beta vulgaris*
Unigene25729_All	4.32	17.55	2.02	*Theobroma cacao*
Unigene508_All	2.57	9.36	1.86	*Glycine max*
Unigene4752_All	4.28	14.78	1.79	*Nelumbo nucifera*
Unigene8975_All	12.37	36.73	1.57	*Pyrus x bretschneideri*
CL3871.Contig2_All	1.59	0.01	-7.31	*Theobroma cacao*
Unigene14311_All	15.82	7.42	-1.09	*Fragaria vesca*
Unigene18009_All	7.25	2.93	-1.31	*Vitis vinifera*
Unigene16844_All	208.08	64.29	-1.69	*Spinacia oleracea*
Unigene5364_All	158.49	41.15	-1.95	*Solanum lycopersicum*
CL4720.Contig2_All	4.38	1.11	-1.98	*Ricinus communis*
CL5758.Contig3_All	2.31	0.17	-3.76	*Nicotiana tomentosiformis*
Unigene11441_All	61.05	13.85	-2.14	*Nelumbo nucifera*

Table S14. Differentially expressed genes (DEGs) related to transcription factor in roots of *A. canescens* after 100 mM NaCl treatment for 6 h. FPKM-CR6 and FPKM-SR6 respectively indicates the FPKM value of a gene in roots under control condition for 6 h and salt treatment for 6 h. Fold change equals to log2 (FPKM-SR6 / FPKM-CR6). Protein refer to the protein encoded by each DEGs.

Gene ID	FPKM-CR6	FPKM-SR6	Fold change	Homologous species
**WRKY**				
CL1071.Contig2_All	193.33	51.73	-1.90	*Spinacia oleracea*
CL2815.Contig2_All	275.99	135.11	-1.03	*Citrus sinensis*
CL3186.Contig2_All	13.02	5.62	-1.21	*Tamarix hispida*
CL3276.Contig1_All	10.67	3.95	-1.43	*Nicotiana tomentosiformis*
CL380.Contig2_All	46.73	2.44	-4.26	*Nelumbo nucifera*
CL3907.Contig1_All	215.52	41.06	-2.39	*Spinacia oleracea*

CL408.Contig2_All	81.79	13.39	-2.61	*Nelumbo nucifera*
CL4275.Contig2_All	37.26	2.05	-4.18	*Vitis vinifera*
CL492.Contig2_All	28.69	3.01	-3.25	*Tamarix hispida*
CL629.Contig1_All	71.2	0.57	-6.96	*Vitis vinifera*
CL7561.Contig1_All	29.45	3.12	-3.24	*Theobroma cacao*
CL7772.Contig1_All	4.51	2.07	-1.12	*Ricinus communis*
Unigene11100_All	6.2	0.06	-6.69	*Nicotiana tomentosiformis*
Unigene12295_All	20.65	2.15	-3.26	*Vitis vinifera*
Unigene1538_All	2.99	0.01	-8.22	*Solanum tuberosum*
Unigene16243_All	14.61	0.01	-10.51	*Theobroma cacao*
Unigene17677_All	9.84	0.72	-3.77	*Panax quinquefolius*
Unigene17816_All	43.46	15.21	-1.51	*Nelumbo nucifera*
Unigene1946_All	10.46	3.87	-1.43	*Tamarix hispida*
Unigene26885_All	38.67	7.52	-2.36	*Tamarix hispida*
Unigene30487_All	3.52	0.01	-8.46	*Glycine max*
Unigene3253_All	47.98	21.01	-1.19	*Vitis aestivalis*
Unigene3615_All	10.12	0.48	-4.40	*Theobroma cacao*
Unigene3697_All	13.79	1.23	-3.49	*Nelumbo nucifera*
Unigene3698_All	62.78	13.46	-2.22	*Theobroma cacao*
Unigene3898_All	4.08	0.53	-2.94	*Nelumbo nucifera*
Unigene40205_All	9	0.01	-9.81	*Theobroma cacao*
Unigene5485_All	20.74	2.83	-2.87	*Populus tomentosa*
Unigene553_All	8.48	1.5	-2.50	*Jatropha curcas*
Unigene5531_All	10.01	0.01	-9.97	*Citrus sinensis*
Unigene6298_All	45.67	16.8	-1.44	*Pyrus x bretschneideri*
Unigene9476_All	10.66	0.07	-7.25	*Vitis vinifera*
Unigene3786_All	1.58	4.68	1.57	*Vitis vinifera*
**MYB**				
CL4105.Contig2_All	8.4	17.9	1.09	*Salicornia brachiata*
Unigene4919_All	1.32	5.55	2.07	*Fragaria vesca subsp. vesca*
Unigene7861_All	2.66	18.14	2.77	*Arachis hypogaea*
Unigene12759_All	0.43	8.59	4.32	*Jatropha curcas*
Unigene3069_All	0.46	26.41	5.84	*Theobroma cacao*
CL1223.Contig2_All	37.56	3.31	-3.50	*Vitis vinifera*
CL2741.Contig4_All	26.5	8.17	-1.70	*Ricinus communis*
CL2987.Contig1_All	8.68	1.43	-2.60	*Ricinus communis*
CL4043.Contig2_All	34.36	0.01	-11.75	*Arachis hypogaea*
CL422.Contig3_All	5.39	0.01	-9.07	*Nelumbo nucifera*
CL4970.Contig1_All	405.42	19.4	-4.39	*Nicotiana tabacum*
CL5105.Contig2_All	4.01	0.6	-2.74	*Populus trichocarpa*
CL5476.Contig1_All	4.93	0.41	-3.59	*Jatropha curcas*
CL5601.Contig1_All	15.42	2.37	-2.70	*Morus notabilis*
CL5951.Contig3_All	7.1	0.01	-9.47	*Quercus suber*
CL7820.Contig1_All	3.38	0.01	-8.40	*Nelumbo nucifera*

CL8721.Contig1_All	7.89	0.01	-9.62	*Populus trichocarpa*
Unigene10405_All	18.19	0.15	-6.92	*Theobroma cacao*
Unigene7714_All	8.91	0.01	-9.80	*Glycine max*
Unigene41594_All	5.92	0.01	-9.21	*Pyrus x bretschneideri*
Unigene28181_All	4.26	0.01	-8.73	*Theobroma cacao*
Unigene403_All	3.04	0.01	-8.25	*Theobroma cacao*
Unigene17573_All	5.3	0.1	-5.73	*Jatropha curcas*
Unigene15334_All	3.44	0.13	-4.73	*Nicotiana tomentosiformis*
Unigene12857_All	34.33	3.3	-3.38	*Populus trichocarpa*
Unigene15802_All	6.64	1.42	-2.23	*Nicotiana sylvestris*
Unigene9243_All	10.18	2.35	-2.12	*Theobroma cacao*
Unigene11056_All	130.36	38.37	-1.76	*Salicornia brachiata*
Unigene10959_All	5.48	1.7	-1.69	*Theobroma cacao*
Unigene7260_All	64.23	23.78	-1.43	*Tamarix hispida*
Unigene13756_All	0.01	2.98	8.22	*Malus domestica*
**bZIP**				
Unigene14640_All	14.68	0.01	-10.52	*Theobroma cacao*
CL1844.Contig2_All	57.99	2.42	-4.58	*Populus trichocarpa*
Unigene221_All	5.59	1.2	-2.22	*Tamarix hispida*
Unigene1814_All	136.55	31.95	-2.10	*Tamarix hispida*
Unigene5773_All	19.62	4.93	-1.99	*Tamarix hispida*
CL4915.Contig1_All	33.51	10.54	-1.67	*Tamarix hispida*
CL6106.Contig3_All	23.93	10.49	-1.19	*Morus notabilis*
Unigene534_All	14.84	5.89	-1.33	*Theobroma cacao*
CL5048.Contig3_All	33.48	3.51	-3.25	*Spinacia oleracea*
Unigene12323_All	1.45	0.01	-7.18	*Prunus mume*
Unigene15896_All	74.4	33.19	-1.16	*Nicotiana tomentosiformis*
Unigene27471_All	1.4	0.01	-7.13	*Nicotiana sylvestris*
Unigene4117_All	4.14	1.38	-1.58	*Theobroma cacao*
Unigene9492_All	0.75	0.01	-6.23	*Theobroma cacao*
CL1962.Contig1_All	21.71	6.4	-1.76	*Vitis vinifera*
CL6157.Contig1_All	3.15	0.52	-2.60	*Vitis vinifera*
Unigene11050_All	2.28	0.01	-7.83	*Nicotiana tomentosiformis*
Unigene12290_All	37.37	4.4	-3.09	*Theobroma cacao*
Unigene145_All	92.26	40.9	-1.17	*Vitis vinifera*
Unigene14582_All	8.18	2.19	-1.90	*Vitis vinifera*
Unigene15839_All	18.65	1.15	-4.02	*Prunus mume*
Unigene16296_All	5.24	0.01	-9.03	*Vitis vinifera*
Unigene3305_All	7.23	3.42	-1.08	*Mirabilis jalapa*
Unigene4320_All	35.73	13.92	-1.36	*Vitis vinifera*
Unigene5448_All	15.52	2.68	-2.53	*Vitis vinifera*
Unigene2090_All	2.06	36.08	4.13	*Solanum tuberosum*
Unigene26259_All	0.24	2.1	3.13	*Prunus mume*
Unigene7774_All	0.08	11.27	7.14	*Vitis vinifera*

Unigene23519_All	0.01	1	6.64	*Vitis vinifera*
Unigene23520_All	0.01	1.04	6.70	*Nicotiana sylvestris*
Unigene26257_All	0.01	3.29	8.36	*Nelumbo nucifera*
**AP2/ERF**				
CL1411.Contig1_All	21.33	0.01	-11.06	*Morus notabilis*
CL2241.Contig1_All	46.94	16.1	-1.54	*Tamarix hispida*
CL3234.Contig2_All	42.86	15.97	-1.42	*Rumex acetosa*
CL4440.Contig1_All	6.15	1.37	-2.17	*Eucalyptus grandis*
CL5582.Contig1_All	28.02	3.29	-3.09	*Citrus sinensis*
CL5907.Contig2_All	6.35	0.01	-9.31	*Catharanthus roseus*
CL7480.Contig2_All	11.38	2.9	-1.97	*Pyrus x bretschneideri*
Unigene10412_All	0.89	0.01	-6.48	*Prunus mume*
Unigene12257_All	7.84	1.56	-2.33	*Nelumbo nucifera*
Unigene13939_All	11.3	4.19	-1.43	*Diospyros kaki*
Unigene14464_All	12.68	3.85	-1.72	*Theobroma cacao*
Unigene14637_All	370.12	80.97	-2.19	*Solanum lycopersicum*
Unigene15713_All	223.32	47.23	-2.24	*Tamarix hispida*
Unigene1741_All	1.39	0.01	-7.12	*Malus domestica*
Unigene17627_All	22.52	3.71	-2.60	*Nelumbo nucifera*
Unigene17680_All	422.19	144.85	-1.54	*Vitis vinifera*
Unigene18392_All	4.67	0.17	-4.78	*Nelumbo nucifera*
Unigene18547_All	16.83	0.69	-4.61	*Theobroma cacao*
Unigene18548_All	3.84	0.01	-8.58	*Theobroma cacao*
Unigene27753_All	6.2	1.04	-2.58	*Nicotiana sylvestris*
Unigene31276_All	22.69	0.01	-11.15	*Pyrus x bretschneideri*
Unigene32394_All	1.96	0.01	-7.61	*Theobroma cacao*
Unigene3600_All	12.59	0.01	-10.30	*Theobroma cacao*
Unigene3750_All	9.14	0.05	-7.51	*Theobroma cacao*
Unigene5762_All	8.6	4.09	-1.07	*Morus notabilis*
Unigene5913_All	47.39	16.21	-1.55	*Solanum lycopersicum*
Unigene6706_All	19.66	2.23	-3.14	*Tamarix hispida*
Unigene6873_All	290.42	133.55	-1.12	*Fragaria vesca subsp. vesca*
Unigene7169_All	12.55	0.39	-5.01	*Solanum tuberosum*
**DREB**				
Unigene15818_All	41.62	1.48	-4.81	*Suaeda salsa*
CL5907.Contig4_All	19.08	3.28	-2.54	*Salicornia brachiata*
Unigene3588_All	91.22	22.65	-2.01	*Salicornia brachiata*
CL6518.Contig1_All	2.89	0.18	-4.01	*Corchorus olitorius*
**NAC**				
CL2948.Contig1_All	4.65	0.45	-3.37	*Theobroma cacao*
CL3.Contig1_All	3.62	1.22	-1.57	*Theobroma cacao*
CL4235.Contig2_All	1.66	0.01	-7.38	*Solanum tuberosum*
CL6151.Contig1_All	8.63	2.47	-1.80	*Malus domestica*

CL7605.Contig3_All	27.04	10.42	-1.38	*Nelumbo nucifera*
CL9208.Contig1_All	27.69	3.07	-3.17	*Morus notabilis*
CL9299.Contig2_All	15.24	7	-1.12	*Jatropha curcas*
Unigene10179_All	81.86	33.62	-1.28	*Suaeda liaotungensis*
Unigene12773_All	7.12	0.76	-3.23	*Hevea brasiliensis*
Unigene1286_All	12.05	0.77	-3.97	*Fragaria vesca subsp. vesca*
Unigene12962_All	4.01	0.71	-2.50	*Pyrus x bretschneideri*
Unigene16456_All	5.67	1.58	-1.84	*Suaeda liaotungensis*
Unigene167_All	17.67	0.01	-10.79	*Theobroma cacao*
Unigene17664_All	33.32	9.1	-1.87	*Phaseolus vulgaris*
Unigene18464_All	8.03	2.85	-1.49	*Nelumbo nucifera*
Unigene18478_All	2.05	0.22	-3.22	*Theobroma cacao*
Unigene31695_All	3.69	0.91	-2.02	*Vitis vinifera*
Unigene3303_All	7.59	1.35	-2.49	*Theobroma cacao*
Unigene34120_All	7.21	0.56	-3.69	*Prunus mume*
Unigene3569_All	10.4	2.41	-2.11	*Theobroma cacao*
Unigene6031_All	8.97	2.3	-1.96	*Nelumbo nucifera*
Unigene6877_All	5.04	0.87	-2.53	*Tamarix hispida*
Unigene17764_All	0.06	13.04	7.76	*Gossypium hirsutum*
**bHLH**				
CL7924.Contig1_All	0.01	4.38	8.77	*Vitis vinifera*
CL3377.Contig1_All	4.03	13.71	1.77	*Vitis vinifera*
CL3710.Contig3_All	2.99	11.1	1.89	*Prunus mume*
CL7707.Contig2_All	0.72	5.88	3.03	*Theobroma cacao*
Unigene8694_All	0.88	43.15	5.62	*Vitis vinifera*
Unigene27729_All	16.75	68.49	2.03	*Theobroma cacao*
CL3156.Contig1_All	38.25	11.8	-1.70	*Malus domestica*
CL4581.Contig2_All	3.53	0.86	-2.04	*Nelumbo nucifera*
CL6102.Contig2_All	31.2	13.41	-1.22	*Vitis vinifera*
CL7559.Contig2_All	14.64	2.85	-2.36	*Theobroma cacao*
CL8001.Contig1_All	5.06	0.68	-2.90	*Vitis vinifera*
CL9026.Contig1_All	9.67	2.52	-1.94	*Theobroma cacao*
CL9130.Contig1_All	3.07	0.01	-8.26	*Citrus sinensis*
CL9504.Contig1_All	9.92	2.67	-1.89	*Vitis vinifera*
Unigene10520_All	8.24	0.01	-9.69	*Lotus japonicus*
Unigene11068_All	21.09	4.76	-2.15	*Theobroma cacao*
Unigene11162_All	5.63	0.2	-4.82	*Solanum lycopersicum*
Unigene12084_All	45.43	2.97	-3.94	*Malus domestica*
Unigene16064_All	28.74	0.14	-7.68	*Prunus mume*
Unigene16138_All	17.04	0.24	-6.15	*Nelumbo nucifera*
Unigene1766_All	13.03	0.01	-10.35	*Nicotiana sylvestris*
Unigene17676_All	9.84	0.55	-4.16	*Fragaria vesca subsp. vesca*
Unigene17695_All	84.78	1.75	-5.60	*Vitis vinifera*
Unigene17775_All	23.22	2.08	-3.48	*Malus domestica*

Unigene20260_All	12.87	3.91	-1.72	*Theobroma cacao*
Unigene27074_All	8.85	4.29	-1.04	*Fragaria vesca subsp. vesca*
Unigene27097_All	8.51	1.64	-2.38	*Theobroma cacao*
Unigene3_All	5.55	2.24	-1.31	*Cicer arietinum*
Unigene3415_All	21.97	8.56	-1.36	*Vitis vinifera*
Unigene4053_All	3.63	0.01	-8.50	*Populus simonii*
Unigene4149_All	4.06	1.51	-1.43	*Vitis vinifera*
Unigene5931_All	10.25	0.01	-10.00	*Vitis vinifera*
Unigene7013_All	3.32	0.01	-8.38	*Vitis vinifera*
Unigene7417_All	3.59	0.51	-2.82	*Vitis vinifera*
Unigene9684_All	64.25	20.49	-1.65	*Vitis vinifera*
**MADS-box**				
CL1478.Contig3_All	0.01	0.65	6.02	*Nelumbo nucifera*
Unigene24561_All	13.17	0.01	-10.36	*Vitis vinifera*
CL6533.Contig2_All	9.39	0.01	-9.87	*Prunus mume*
Unigene9423_All	9.06	0.01	-9.82	*Nicotiana tomentosiformis*
CL5411.Contig2_All	0.42	0.01	-5.39	*Nelumbo nucifera*
CL2234.Contig5_All	3.75	0.01	-8.55	*Triticum aestivum*
Unigene10524_All	5	2.31	-1.11	*Populus tremuloides*
Unigene23053_All	1.32	0.01	-7.04	*Theobroma cacao*
Unigene31802_All	0.22	0.01	-4.46	*Nicotiana tomentosiformis*
**ZF**				
Unigene8363_All	3.26	0.01	-8.35	*Theobroma cacao*
CL1528.Contig2_All	6.75	0.63	-3.42	*Malus domestica*
CL7870.Contig2_All	5.74	0.85	-2.76	*Eucalyptus grandis*
CL3079.Contig2_All	4.2	1.05	-2.00	*Cucumis melo*
CL6093.Contig1_All	7.04	2.15	-1.71	*Theobroma cacao*
CL104.Contig19_All	15.99	4.16	-1.94	*Pyrus x bretschneideri*
CL1059.Contig5_All	7.83	3.82	-1.04	*Morus notabilis*
CL1153.Contig1_All	6.48	2.71	-1.26	*Vitis vinifera*
CL1249.Contig2_All	7.45	2.24	-1.73	*Prunus mume*
CL1456.Contig3_All	12.81	4.94	-1.37	*Phoenix dactylifera*
CL1525.Contig2_All	3.17	0.97	-1.71	*Linum usitatissimum*
CL213.Contig2_All	5.91	2.1	-1.49	*Brassica rapa*
CL2230.Contig2_All	7.89	2.91	-1.44	*Vitis vinifera*
CL2251.Contig1_All	3.65	0.52	-2.81	*Prunus mume*
CL2680.Contig1_All	8.59	2.92	-1.56	*Pyrus x bretschneideri*
CL2784.Contig1_All	14.65	6.94	-1.08	*Solanum lycopersicum*
CL2904.Contig5_All	9.94	1.49	-2.74	*Citrus sinensis*
CL3061.Contig1_All	73.81	0.01	-12.85	*Theobroma cacao*
CL3473.Contig1_All	2.01	0.01	-7.65	*Nicotiana sylvestris*
CL3528.Contig2_All	4.47	1.87	-1.26	*Nicotiana sylvestris*
CL5822.Contig2_All	3.15	0.92	-1.78	*Arabidopsis thaliana*
CL5844.Contig3_All	4.27	0.01	-8.74	*Theobroma cacao*

CL6565.Contig2_All	3.21	0.01	-8.33	*Vitis vinifera*
CL6912.Contig1_All	15.87	0.37	-5.42	*Citrus sinensis*
CL3811.Contig4_All	33.86	5.05	-2.75	*Nicotiana sylvestris*
CL6239.Contig1_All	40.09	6.27	-2.68	*Glycine max*
CL6005.Contig1_All	3.91	0.77	-2.34	*Theobroma cacao*
CL647.Contig3_All	18.41	4.25	-2.11	*Nelumbo nucifera*
CL7175.Contig2_All	47.35	11.12	-2.09	*Nelumbo nucifera*
CL4249.Contig1_All	74.07	18.3	-2.02	*Populus trichocarpa*
CL6108.Contig1_All	13.67	3.66	-1.90	*Glycine max*
CL5085.Contig3_All	1.23	0.33	-1.90	*Theobroma cacao*
CL7205.Contig2_All	14.33	3.97	-1.85	*Beta vulgaris subsp. vulgaris*
CL4024.Contig2_All	16.74	5.36	-1.64	*Malus domestica*
CL4800.Contig1_All	9.92	3.96	-1.32	*Theobroma cacao*
CL4998.Contig3_All	9.85	3.98	-1.31	*Nicotiana tomentosiformis*
CL6754.Contig1_All	4.83	2.35	-1.04	*Theobroma cacao*
CL7346.Contig1_All	22.19	4.76	-2.22	*Pyrus x bretschneideri*
CL7398.Contig1_All	67.54	23.5	-1.52	*Theobroma cacao*
CL7548.Contig1_All	7.05	2.13	-1.73	*Nelumbo nucifera*
CL7981.Contig1_All	7.18	2.88	-1.32	*Medicago truncatula*
CL8054.Contig1_All	31.05	6.75	-2.20	*Nicotiana tomentosiformis*
CL8254.Contig1_All	12.04	4.5	-1.42	*Theobroma cacao*
CL8476.Contig1_All	8.01	1.86	-2.11	*Nicotiana tomentosiformis*
CL8626.Contig3_All	14.28	6.95	-1.04	*Nelumbo nucifera*
Unigene10303_All	4.22	1.04	-2.02	*Theobroma cacao*
Unigene10320_All	6.54	2.72	-1.27	*Theobroma cacao*
Unigene10801_All	6.55	0.28	-4.55	*Solanum tuberosum*
Unigene11037_All	14.1	4.93	-1.52	*Silene latifolia*
Unigene11889_All	3.58	0.01	-8.48	*Ricinus communis*
Unigene12056_All	26.98	10.88	-1.31	*Theobroma cacao*
Unigene12085_All	24.99	5.16	-2.28	*Ricinus communis*
Unigene1234_All	31.04	1.03	-4.91	*Populus trichocarpa*
Unigene12834_All	29.07	4.36	-2.74	*Vitis vinifera*
Unigene13066_All	48.32	23.67	-1.03	*Populus trichocarpa*
Unigene1344_All	1.78	0.01	-7.48	*Brassica rapa*
Unigene13958_All	8.34	3.51	-1.25	*Nelumbo nucifera*
Unigene1451_All	26.12	6.26	-2.06	*Vitis vinifera*
Unigene1480_All	106.72	4.4	-4.60	*Eucalyptus grandis*
Unigene18007_All	40.36	0.01	-11.98	*Nicotiana sylvestris*
Unigene30323_All	5.23	0.01	-9.03	*Nicotiana tomentosiformis*
Unigene18411_All	3.77	0.01	-8.56	*Malus domestica*
Unigene6975_All	31.33	0.44	-6.15	*Morus notabilis*
Unigene15982_All	37.27	1.32	-4.82	*Populus trichocarpa*
Unigene35464_All	6.56	0.53	-3.63	*Solanum lycopersicum*
Unigene461_All	4.2	0.37	-3.50	*Nelumbo nucifera*

Unigene251_All	2.55	0.26	-3.29	*Medicago truncatula*
Unigene31278_All	16.75	1.94	-3.11	*Glycine max*
Unigene16195_All	9.17	1.91	-2.26	*Pyrus x bretschneideri*
Unigene3903_All	4.1	0.94	-2.12	*Nelumbo nucifera*
Unigene8602_All	9.06	2.59	-1.81	*Nelumbo nucifera*
Unigene27507_All	4.1	1.33	-1.62	*Nicotiana sylvestris*
Unigene268_All	45.55	16.62	-1.45	*Nicotiana sylvestris*
Unigene17840_All	27.73	10.71	-1.37	*Morus notabilis*
Unigene6014_All	9.93	3.96	-1.33	*Medicago truncatula*
Unigene18146_All	9.85	3.99	-1.30	*Vitis vinifera*
Unigene5212_All	10.69	4.42	-1.27	*Vitis vinifera*
Unigene2389_All	15.41	6.38	-1.27	*Vitis vinifera*
Unigene8686_All	20.36	8.87	-1.20	*Theobroma cacao*
Unigene4926_All	13.7	6.18	-1.15	*Prunus mume*
Unigene5790_All	7.3	3.42	-1.09	*Theobroma cacao*
Unigene16891_All	20.26	9.98	-1.02	*Prunus mume*
Unigene6232_All	17.56	8.74	-1.01	*Ricinus communis*
CL2153.Contig3_All	1.7	4.94	1.54	*Theobroma cacao*
CL7243.Contig1_All	9.54	21.92	1.20	*Nelumbo nucifera*
CL7243.Contig3_All	2.47	8.1	1.71	*Nelumbo nucifera*
CL8626.Contig2_All	1.61	9.77	2.60	*Nelumbo nucifera*
CL9583.Contig2_All	0.1	12.72	6.99	*Saruma henryi*
CL5689.Contig2_All	2.51	16.84	2.75	*Theobroma cacao*
Unigene7177_All	4.6	18.11	1.98	*Vitis vinifera*
Unigene12010_All	10.82	34.4	1.67	*Prunus mume*
Unigene7539_All	1.39	3.99	1.52	*Theobroma cacao*
CL5483.Contig2_All	39.11	82.06	1.07	*Theobroma cacao*
Unigene16749_All	12.9	27.06	1.07	*Theobroma cacao*
Unigene8812_All	24.39	53.84	1.14	*Nicotiana sylvestris*
Unigene18264_All	8.47	21.69	1.36	*Populus trichocarpa*
Unigene5811_All	3.33	8.82	1.41	*Prunus mume*
CL8887.Contig1_All	1.37	5.38	1.97	*Medicago truncatula*
Unigene12475_All	0.7	6.77	3.27	*Medicago truncatula*
CL5054.Contig1_All	4.2	13.07	1.64	*Cicer arietinum*
CL6271.Contig1_All	1.16	4.83	2.06	*Populus trichocarpa*
CL5550.Contig2_All	5.71	31.99	2.49	*Glycine max*
CL4536.Contig3_All	1.88	10.94	2.54	*Solanum lycopersicum*
CL4442.Contig2_All	4.18	29.03	2.80	*Vitis vinifera*
Unigene5674_All	0.01	1.85	7.53	*Prunus mume*
CL2069.Contig1_All	0.01	18.51	10.85	*Nelumbo nucifera*
CL4799.Contig2_All	0.01	1.61	7.33	*Pyrus x bretschneideri*
CL37.Contig3_All	0.01	1.92	7.58	*Populus trichocarpa*
CL6690.Contig2_All	0.01	79.44	12.96	*Vitis vinifera*
**HSF**				

CL1745.Contig1_All	12.4	5.14	-1.27	*Populus trichocarpa*
CL1745.Contig2_All	32.55	11.15	-1.55	*Populus trichocarpa*
CL1986.Contig1_All	2.95	0.23	-3.68	*Capparis spinosa*
CL1986.Contig3_All	27.74	7.03	-1.98	*Solanum tuberosum*
CL1986.Contig4_All	25.54	8.37	-1.61	*Vitis vinifera*
CL2066.Contig3_All	17.16	5.87	-1.55	*Citrus sinensis*
CL2066.Contig4_All	4.43	1.04	-2.09	*Musa acuminata*
CL2779.Contig1_All	6.99	1.52	-2.20	*Vitis vinifera*
CL2779.Contig2_All	5.24	1.77	-1.57	*Vitis vinifera*
CL3372.Contig1_All	26	0.91	-4.84	*Cucumis melo*
CL3372.Contig2_All	28.41	0.01	-11.47	*Cucumis melo*
CL5603.Contig1_All	3.37	0.82	-2.04	*Ricinus communis*
CL6191.Contig2_All	11.25	1.13	-3.32	*Chenopodium album*
CL6296.Contig1_All	44.76	6.85	-2.71	*Oxybasis rubra*
CL6546.Contig1_All	2.77	0.01	-8.11	*Theobroma cacao*
CL6546.Contig5_All	2.29	0.37	-2.63	*Theobroma cacao*
CL7113.Contig1_All	7.89	1.15	-2.78	*Cucumis melo*
CL7682.Contig1_All	13.18	0.17	-6.28	*Vitis vinifera*
CL7682.Contig2_All	8.5	0.01	-9.73	*Vitis vinifera*
CL8208.Contig5_All	5.84	1.57	-1.90	*Vitis vinifera*
Unigene29093_All	5.54	0.01	-9.11	*Micromonas pusilla CCMP1545*
Unigene18326_All	24.33	0.36	-6.08	*Nicotiana tomentosiformis*
Unigene5261_All	42.95	2.91	-3.88	*Pyrus x bretschneideri*
Unigene10400_All	22.69	4.35	-2.38	*Medicago truncatula*
Unigene12840_All	6.33	1.5	-2.08	*Nicotiana tomentosiformis*
Unigene4752_All	10.69	2.67	-2.00	*Nelumbo nucifera*
Unigene3969_All	8.76	2.27	-1.95	*Beta vulgaris*
Unigene1293_All	16.75	5.02	-1.74	*Rosa hybrid cultivar*
Unigene8975_All	47.71	15.78	-1.60	*Pyrus x bretschneideri*
Unigene18261_All	11.7	4.37	-1.42	*Glycine max*
Unigene508_All	7.74	3	-1.37	*Glycine max*
Unigene25729_All	9.26	3.86	-1.26	*Theobroma cacao*
Unigene7332_All	3.96	1.81	-1.13	*Ricinus communis*
Unigene17363_All	11.08	5.25	-1.08	*Setaria italica*
Unigene4936_All	14.53	7.05	-1.04	*Theobroma cacao*
Unigene5364_All	50.04	105.32	1.07	*Solanum lycopersicum*
Unigene11441_All	16.44	60.35	1.88	*Nelumbo nucifera*
Unigene16844_All	58.19	231.61	1.99	*Spinacia oleracea*
CL5758.Contig3_All	0.88	2.93	1.74	*Nicotiana tomentosiformis*
CL7449.Contig2_All	11.7	49.47	2.08	*Chenopodium album*
CL3871.Contig2_All	0.01	1.69	7.40	*Theobroma cacao*

Table S15. Differentially expressed genes (DEGs) related to protein kinase in leaves of *A. canescens* after 100 mM NaCl treatment for 6 h. FPKM-CL6 and FPKM-SL6 respectively indicates the FPKM value of a gene in leaves under control condition for 6 h and salt treatment

for 6 h. Fold change equals to log2 (FPKM-SL6 / FPKM-CL6). Protein refer to the protein encoded by each DEGs.

Gene ID	FPKM -CL6	FPKM -SL6	Fold change	Homologous species
**RLK**				
Unigene35208_All	1.18	0.01	-6.88	*Vitis vinifera*
Unigene36621_All	0.62	0.01	-5.95	*Eucalyptus grandis*
CL4075.Contig1_All	2.71	0.54	-2.32	*Solanum tuberosum*
Unigene2959_All	5.49	1.29	-2.09	*Solanum tuberosum*
CL4830.Contig1_All	1.82	0.45	-2.02	*Vitis vinifera*
Unigene7148_All	6.28	1.86	-1.76	*Nelumbo nucifera*
Unigene15443_All	1.02	0.51	-1.00	*Morus notabilis*
Unigene13701_All	37.7	0.4	-6.56	*Brassica rapa*
Unigene39455_All	0.67	0.01	-6.07	*Arabidopsis thaliana*
CL6747.Contig2_All	0.59	0.01	-5.88	*Phoenix dactylifera*
Unigene24849_All	0.41	0.01	-5.36	*Phoenix dactylifera*
CL1768.Contig7_All	6.67	0.29	-4.52	*Prunus mume*
Unigene29556_All	0.77	0.26	-1.57	*Malus domestica*
Unigene11654_All	13.82	42.36	1.62	*Nicotiana sylvestris*
Unigene7507_All	1.43	5.62	1.97	*Morus notabilis*
CL1604.Contig3_All	3.32	13.81	2.06	*Vitis vinifera*
CL3685.Contig2_All	0.03	5.42	7.50	*Ricinus communis*
Unigene12732_All	0.27	8.7	5.01	*Ricinus communis*
Unigene16763_All	5.96	12.16	1.03	*Prunus mume*
Unigene34017_All	0.65	2.21	1.77	*Nelumbo nucifera*
Unigene30033_All	1.48	6.67	2.17	*Musa acuminata*
Unigene9831_All	0.8	7.42	3.21	*Nicotiana tomentosiformis*
Unigene13978_All	0.32	70.05	7.77	*Pyrus x bretschneideri*
Unigene15867_All	0.62	6.09	3.30	*Vitis vinifera*
CL3305.Contig3_All	0.48	3.28	2.77	*Vitis vinifera*
CL2170.Contig4_All	0.79	4.81	2.60	*Prunus mume*
CL2170.Contig5_All	1.35	6.43	2.25	*Nelumbo nucifera*
Unigene14470_All	1.41	6.28	2.15	*Vitis vinifera*
CL7493.Contig2_All	2.23	20.57	3.21	*Nelumbo nucifera*
Unigene17553_All	2.2	6.66	1.59	*Prunus mume*
Unigene16822_All	7.13	20.88	1.55	*Nelumbo nucifera*
Unigene8447_All	15.13	31.07	1.04	*Vitis vinifera*
CL264.Contig2_All	13.04	55.21	2.08	*Vitis vinifera*
CL2170.Contig3_All	0.01	0.21	4.39	*Morus notabilis*
CL2893.Contig5_All	0.01	0.64	6.00	*Nelumbo nucifera*
Unigene38755_All	0.01	0.68	6.08	*Nelumbo nucifera*
CL7451.Contig5_All	0.01	0.7	6.13	*Phoenix dactylifera*
Unigene33490_All	0.01	1.04	6.70	*Pyrus x bretschneideri*
CL6175.Contig1_All	0.01	1.33	7.06	*Vitis vinifera*

Unigene34019_All	0.01	2.22	7.79	*Vitis vinifera*
Unigene36094_All	0.01	3.2	8.32	*Nelumbo nucifera*
Unigene21989_All	0.01	15.35	10.58	*Nelumbo nucifera*
Unigene1080_All	0.01	0.28	4.80	*Solanum lycopersicum*
Unigene30353_All	0.01	1.38	7.10	*Ricinus communis*
**WAK**				
CL7712.Contig2_All	4.43	0.01	-8.79	*Cucumis sativus*
Unigene2643_All	4.07	0.01	-8.67	*Theobroma cacao*
Unigene4677_All	2.52	0.01	-7.98	*Ricinus communis*
Unigene22036_All	1.67	0.01	-7.38	*Ricinus communis*
CL1625.Contig4_All	0.66	0.01	-6.04	*Solanum lycopersicum*
CL171.Contig1_All	0.65	0.01	-6.02	*Medicago truncatula*
Unigene4114_All	6.9	0.17	-5.34	*Prunus mume*
Unigene8224_All	3.11	0.18	-4.11	*Vitis vinifera*
Unigene20305_All	2.71	0.34	-2.99	*Citrus sinensis*
CL342.Contig2_All	2.85	0.54	-2.40	*Eucalyptus grandis*
CL2993.Contig2_All	5.02	1	-2.33	*Fragaria vesca*
Unigene4137_All	3.27	1.35	-1.28	*Nelumbo nucifera*
Unigene11429_All	13.07	32	1.29	*Eucalyptus grandis*
Unigene5799_All	1.51	4.06	1.43	*Vitis vinifera*
Unigene3955_All	2.93	8.36	1.51	*Nicotiana tomentosiformis*
Unigene15340_All	0.71	2.14	1.59	*Nicotiana sylvestris*
CL1625.Contig3_All	0.35	1.06	1.60	*Zea mays*
CL6066.Contig1_All	3.58	10.87	1.60	*Ricinus communis*
CL219.Contig9_All	1.26	4.62	1.87	*Eucalyptus grandis*
Unigene732_All	1.44	5.53	1.94	*Nicotiana tabacum*
Unigene29407_All	0.41	1.63	1.99	*Vitis vinifera*
CL219.Contig3_All	2.56	10.32	2.01	*Citrus sinensis*
CL1625.Contig1_All	1.02	4.44	2.12	*Nelumbo nucifera*
CL219.Contig7_All	1.78	8.71	2.29	*Nelumbo nucifera*
CL219.Contig10_All	1.61	8.83	2.45	*Citrus sinensis*
Unigene4688_All	0.64	3.85	2.59	*Citrus sinensis*
CL219.Contig12_All	1.79	13.41	2.90	*Eucalyptus grandis*
Unigene36213_All	0.26	2.34	3.17	*Cucumis melo*
Unigene16011_All	2.53	35.89	3.83	*Nelumbo nucifera*
Unigene850_All	0.26	6.18	4.57	*Ricinus communis*
CL7712.Contig1_All	0.42	24.1	5.84	*Nelumbo nucifera*
CL7712.Contig3_All	0.29	20.35	6.13	*Nelumbo nucifera*
Unigene36859_All	0.01	0.72	6.16	*Solanum lycopersicum*
Unigene29472_All	0.01	1.24	6.95	*Nicotiana sylvestris*
Unigene34082_All	0.01	1.78	7.48	*Morus notabilis*
CL8521.Contig2_All	0.01	2.47	7.95	*Eucalyptus grandis*
Unigene35516_All	0.01	3.54	8.47	*Nelumbo nucifera*

LecRLK				
Unigene9770_All	5.95	0.01	-9.22	*Prunus mume*
CL9096.Contig1_All	2.64	0.01	-8.04	*Pyrus x bretschneideri*
CL6802.Contig1_All	1.94	0.01	-7.60	*Eucalyptus grandis*
CL30.Contig9_All	1.46	0.01	-7.19	*Eucalyptus grandis*
CL126.Contig1_All	0.53	0.01	-5.73	*Brassica rapa*
Unigene16277_All	17.32	0.36	-5.58	*Cicer arietinum*
CL7244.Contig2_All	0.36	0.01	-5.17	*Nelumbo nucifera*
Unigene40469_All	0.36	0.01	-5.17	*Populus trichocarpa*
Unigene2748_All	1.28	0.14	-3.19	*Vitis vinifera*
CL4407.Contig1_All	5.86	1.18	-2.31	*Malus domestica*
Unigene22602_All	4.7	1.34	-1.81	*Brassica rapa*
CL1876.Contig5_All	5.01	1.56	-1.68	*Vitis vinifera*
CL2621.Contig4_All	7.87	2.81	-1.48	*Pyrus x bretschneideri*
CL1044.Contig2_All	4.66	1.83	-1.35	*Vitis vinifera*
CL3313.Contig6_All	0.39	0.78	1.00	*Malus domestica*
Unigene12686_All	1.62	3.29	1.02	*Vitis vinifera*
CL6941.Contig1_All	18.83	38.83	1.04	*Theobroma cacao*
CL2927.Contig5_All	0.11	0.24	1.13	*Glycine max*
Unigene7995_All	0.41	1	1.29	*Nelumbo nucifera*
CL2927.Contig2_All	2.33	6.66	1.51	*Cucumis sativus*
CL5713.Contig2_All	2.02	5.78	1.52	*Malus domestica*
CL2011.Contig2_All	1.84	5.37	1.54	*Nicotiana sylvestris*
CL3763.Contig3_All	1.15	3.37	1.55	*Nelumbo nucifera*
Unigene35065_All	0.43	1.3	1.60	*Glycine max*
CL136.Contig1_All	3.5	12.18	1.79	*Nicotiana tomentosiformis*
Unigene12173_All	2.57	9.11	1.82	*Nelumbo nucifera*
Unigene35732_All	0.56	2.25	2.00	*Fragaria vesca*
CL2927.Contig8_All	0.5	2.01	2.00	*Glycine max*
Unigene14931_All	1.3	5.64	2.11	*Prunus mume*
Unigene114_All	0.4	1.86	2.22	*Vitis vinifera*
CL2927.Contig1_All	2.03	9.59	2.24	*Cucumis sativus*
CL8882.Contig1_All	1.62	9.14	2.50	*Citrus sinensis*
CL2011.Contig1_All	2.67	15.23	2.51	*Citrus sinensis*
CL1044.Contig1_All	0.58	3.5	2.59	*Cucumis melo*
Unigene12763_All	1.2	7.56	2.66	*Theobroma cacao*
Unigene14705_All	0.43	2.81	2.71	*Vitis vinifera*
CL1876.Contig2_All	2.89	21.27	2.88	*Vitis vinifera*
CL6552.Contig6_All	0.97	7.39	2.93	*Glycine max*
Unigene16516_All	0.45	6	3.74	*Brassica rapa*
Unigene27493_All	0.36	5.19	3.85	*Vitis vinifera*
CL389.Contig2_All	0.76	21.55	4.83	*Vitis vinifera*
CL1876.Contig4_All	1.15	42.64	5.21	*Vitis vinifera*
CL1876.Contig1_All	0.58	24.64	5.41	*Vitis vinifera*

Unigene3944_All	0.09	4.42	5.62	*Vitis vinifera*
Unigene14677_All	0.22	15.83	6.17	*Theobroma cacao*
Unigene21219_All	0.01	0.58	5.86	*Nelumbo nucifera*
Unigene26968_All	0.01	1.03	6.69	*Nicotiana tomentosiformis*
Unigene37389_All	0.01	1.43	7.16	*Nicotiana sylvestris*
Unigene34574_All	0.01	2.69	8.07	*Citrus sinensis*
CL3763.Contig1_All	0.01	3.18	8.31	*Nelumbo nucifera*
CL7452.Contig2_All	0.01	7.74	9.60	*Vitis vinifera*
Unigene18214_All	0.01	44.59	12.12	*Eucalyptus grandis*
LRR-RLK				
CL1803.Contig2_All	9.77	0.01	-9.93	*Nelumbo nucifera*
CL5423.Contig2_All	7.87	0.01	-9.62	*Nicotiana tomentosiformis*
CL1231.Contig3_All	5.82	0.01	-9.18	*Theobroma cacao*
CL1231.Contig1_All	4.97	0.01	-8.96	*Theobroma cacao*
Unigene13207_All	4.75	0.01	-8.90	*Vitis vinifera*
CL6713.Contig1_All	1.71	0.01	-7.42	*Solanum lycopersicum*
CL8833.Contig2_All	1.19	0.01	-6.89	*Eucalyptus grandis*
Unigene26697_All	1.17	0.01	-6.87	*Vitis vinifera*
CL7338.Contig1_All	0.98	0.01	-6.61	*Silene latifolia*
Unigene35162_All	0.84	0.01	-6.39	*Eucalyptus grandis*
Unigene7808_All	17.17	0.33	-5.70	*Vitis vinifera*
CL1803.Contig3_All	14.91	0.39	-5.25	*Nelumbo nucifera*
CL9137.Contig2_All	7.09	0.24	-4.88	*Vitis vinifera*
CL1803.Contig5_All	35.93	1.92	-4.22	*Malus domestica*
CL1803.Contig1_All	29.3	1.63	-4.17	*Eucalyptus grandis*
CL5423.Contig3_All	14.27	1.11	-3.68	*Nicotiana tomentosiformis*
Unigene7620_All	3.31	0.29	-3.51	*Morus notabilis*
CL1803.Contig4_All	21.36	3.34	-2.67	*Eucalyptus grandis*
CL1729.Contig3_All	1.09	0.28	-1.96	*Musa acuminata*
Unigene15613_All	11.89	3.29	-1.85	*Populus trichocarpa*
CL8233.Contig1_All	2.01	0.61	-1.72	*Vitis vinifera*
CL974.Contig1_All	3.43	1.08	-1.67	*Citrus sinensis*
CL5874.Contig2_All	6.27	2.04	-1.62	*Theobroma cacao*
Unigene3742_All	4.3	1.83	-1.23	*Vitis vinifera*
Unigene13104_All	2.82	5.64	1.00	*Theobroma cacao*
Unigene30007_All	0.26	0.53	1.02	*Pyrus x bretschneideri*
CL7879.Contig2_All	7.58	15.76	1.05	*Prunus mume*
CL1909.Contig2_All	3.19	6.84	1.10	*Nicotiana tomentosiformis*
CL4514.Contig2_All	6.46	14.35	1.15	*Solanum lycopersicum*
CL7967.Contig2_All	2.34	5.25	1.16	*Nicotiana sylvestris*
CL1729.Contig1_All	0.24	0.54	1.16	*Vitis vinifera*
CL9025.Contig1_All	0.98	2.24	1.19	*Cicer arietinum*
Unigene16745_All	12.83	29.7	1.21	*Theobroma cacao*
CL7365.Contig2_All	1.72	4.45	1.37	*Vitis vinifera*

CL652.Contig2_All	7.62	20.14	1.40	*Nelumbo nucifera*
CL1483.Contig1_All	6.64	17.69	1.41	*Nicotiana sylvestris*
CL7879.Contig1_All	5.78	15.49	1.42	*Prunus mume*
Unigene2462_All	36.35	105.75	1.54	*Prunus mume*
Unigene35817_All	0.57	1.73	1.60	*Vitis vinifera*
Unigene3321_All	4.11	12.76	1.63	*Vitis vinifera*
CL974.Contig2_All	1.46	4.61	1.66	*Citrus sinensis*
Unigene1518_All	6.14	20.11	1.71	*Malus domestica*
CL4956.Contig2_All	1.17	4.5	1.94	*Vitis vinifera*
Unigene13054_All	0.91	3.54	1.96	*Vitis vinifera*
Unigene4286_All	2.63	12.44	2.24	*Vitis vinifera*
Unigene6674_All	3.25	17.01	2.39	*Prunus mume*
CL7881.Contig2_All	1.72	9.18	2.42	*Pyrus x bretschneideri*
Unigene11412_All	0.69	4.12	2.58	*Pyrus x bretschneideri*
CL2084.Contig1_All	8.83	59.7	2.76	*Glycine max*
Unigene39769_All	0.13	1.39	3.42	*Vitis vinifera*
Unigene10870_All	0.03	3.38	6.82	*Morus notabilis*
Unigene1911_All	0.03	7.35	7.94	*Prunus mume*
Unigene6058_All	3.91	8.16	1.06	*Ricinus communis*
Unigene12904_All	1.93	4.08	1.08	*Vitis vinifera*
Unigene6664_All	6.62	14.65	1.15	*Citrus sinensis*
Unigene29730_All	0.71	1.65	1.22	*Cucumis sativus*
Unigene11487_All	17.12	42.03	1.30	*Vitis vinifera*
Unigene4127_All	2.75	7.91	1.52	*Theobroma cacao*
Unigene32199_All	1.7	5.09	1.58	*Setaria italica*
Unigene5546_All	1.33	4.24	1.67	*Nicotiana sylvestris*
Unigene17179_All	4.77	17.21	1.85	*Vitis vinifera*
Unigene11535_All	4.18	16.1	1.95	*Morus notabilis*
CL2928.Contig4_All	0.93	5.68	2.61	*Nelumbo nucifera*
Unigene11338_All	0.68	4.24	2.64	*Pyrus x bretschneideri*
Unigene10979_All	1.38	9.64	2.80	*Arabidopsis thaliana*
CL2084.Contig2_All	9.2	70.4	2.94	*Vitis vinifera*
Unigene6612_All	2.71	22.62	3.06	*Theobroma cacao*
Unigene239_All	2.06	19.1	3.21	*Nicotiana tomentosiformis*
Unigene163_All	0.25	2.53	3.34	*Vitis vinifera*
CL8471.Contig1_All	0.19	2.02	3.41	*Theobroma cacao*
Unigene29740_All	0.2	3.11	3.96	*Theobroma cacao*
Unigene16398_All	1.46	28.94	4.31	*Theobroma cacao*
Unigene5661_All	0.35	7.32	4.39	*Nelumbo nucifera*
Unigene11029_All	0.08	1.71	4.42	*Populus trichocarpa*
CL2928.Contig5_All	0.46	9.98	4.44	*Nelumbo nucifera*
Unigene12746_All	0.05	2.3	5.52	*Pyrus x bretschneideri*
Unigene162_All	0.11	13.71	6.96	*Theobroma cacao*
CL657.Contig3_All	10.37	43.45	2.07	*Nicotiana tomentosiformis*

Unigene38560_All	0.01	0.8	6.32	*Cicer arietinum*
Unigene31266_All	0.01	1.04	6.70	*Ricinus communis*
Unigene36355_All	0.01	1.38	7.11	*Solanum tuberosum*
Unigene38082_All	0.01	1.39	7.12	*Zea mays*
Unigene2037_All	0.01	4.28	8.74	*Theobroma cacao*
Unigene12747_All	0.01	6.69	9.39	*Nicotiana tomentosiformis*
CL2166.Contig3_All	0.01	21.72	11.08	*Vitis vinifera*
CL2166.Contig1_All	0.01	31.9	11.64	*Vitis vinifera*
Unigene39549_All	0.01	0.95	6.57	*Vitis vinifera*
Unigene31458_All	0.01	1.32	7.04	*Eucalyptus grandis*
CL1768.Contig6_All	0.01	1.53	7.26	*Vitis vinifera*
Unigene30665_All	0.01	2.62	8.03	*Fragaria vesca*
CL371.Contig3_All	0.01	2.85	8.15	*Prunus mume*
Unigene37147_All	0.01	4.38	8.77	*Nicotiana tomentosiformis*
Unigene14583_All	0.01	5.73	9.16	*Malus domestica*
CL6713.Contig2_All	0.01	8.58	9.74	*Solanum lycopersicum*
Unigene450_All	0.01	9.78	9.93	*Nelumbo nucifera*
CL2166.Contig2_All	0.01	26.87	11.39	*Solanum tuberosum*
Unigene12036_All	0.01	65.55	12.68	*Fragaria vesca*
LysM				
Unigene40057_All	0.53	0.01	-5.73	*Pyrus x bretschneideri*
CL6066.Contig2_All	0.19	5.27	4.79	*Nelumbo nucifera*
CL4753.Contig2_All	1.64	3.28	1.00	*Nelumbo nucifera*
Unigene28415_All	0.01	2.98	8.22	*Cercis chinensis*
CDPK				
CL280.Contig5_All	3.13	0.01	-8.29	*Theobroma cacao*
Unigene22774_All	0.94	0.01	-6.55	*Ricinus communis*
CL280.Contig7_All	5.48	0.33	-4.05	*Theobroma cacao*
CL280.Contig6_All	4.31	0.78	-2.47	*Theobroma cacao*
Unigene7837_All	20.4	75.41	1.89	*Vitis vinifera*
CL5216.Contig2_All	2.96	13.5	2.19	*Mesembryanthemum crystallinum*
CL4209.Contig1_All	0.65	4.03	2.63	*Theobroma cacao*
Unigene9182_All	5.37	34.46	2.68	*Theobroma cacao*
CL8340.Contig1_All	1.28	15.8	3.63	*Theobroma cacao*
CL8340.Contig1_All	21.09	82.66	1.97	*Beta vulgaris*
MAPK				
CL1892.Contig1_All	32.64	0.01	-11.67	*Vitis vinifera*
CL1892.Contig2_All	13.05	0.01	-10.35	*Vitis vinifera*
Unigene14008_All	4.63	0.22	-4.40	*Vitis vinifera*
CL7275.Contig1_All	7.25	3	-1.27	*Chenopodium album*
Unigene16491_All	2.22	0.95	-1.22	*Erythranthe guttata*
Unigene5680_All	4.21	8.78	1.06	*Prunus mume*
CL7275.Contig3_All	13.71	28.64	1.06	*Chenopodium album*

Unigene8625_All	26.17	60.65	1.21	*Rheum australe*
CL8162.Contig1_All	6.38	16.55	1.38	*Vitis vinifera*
Unigene3216_All	26.25	88.37	1.75	*Prunus persica*
Unigene22967_All	5.59	27.99	2.32	*Coffea canephora*
CL4776.Contig2_All	0.79	6.39	3.02	*Vitis vinifera*
Unigene10295_All	0.39	3.33	3.09	*Phaseolus vulgaris*
Unigene10696_All	2.51	24.28	3.27	*Citrus clementina*
Unigene30352_All	0.01	4.42	8.79	*Fragaria vesca*
**CTR1**				
CL9787.Contig2_All	54.27	12.38	-2.13	*Celosia argentea*
CL4515.Contig2_All	1.09	14.59	3.74	*Vitis vinifera*
CL6621.Contig2_All	9.17	29.82	1.70	*Vitis vinifera*
CL4515.Contig1_All	1.31	7.03	2.42	*Vitis vinifera*
casein kinase				
CL2804.Contig4_All	0.01	4.3	8.75	*Vitis vinifera*
Unigene15130_All	19.05	42.97	1.17	*Beta vulgaris*

Table S16. Differentially expressed genes (DEGs) related to protein kinase in leaves of *A. canescens* after 100 mM NaCl treatment for 24 h. FPKM-CL24 and FPKM-SL24 respectively indicates the FPKM value of a gene in leaves under control condition for 24 h and salt treatment for 24 h. Fold change equals to log2 (FPKM-SL24 / FPKM-CL24). Protein refer to the protein encoded by each DEGs.

Gene ID	FPKM-CL24	FPKM-SL24	Fold change	Homologous species
**RLK**				
Unigene10117_All	0.57	0.01	-5.83	*Theobroma cacao*
Unigene16698_All	1.11	0.01	-6.79	*Prunus mume*
CL6747.Contig2_All	0.85	0.01	-6.41	*Phoenix dactylifera*
Unigene36094_All	0.43	0.01	-5.43	*Nelumbo nucifera*
Unigene24849_All	0.38	0.01	-5.25	*Phoenix dactylifera*
CL7493.Contig1_All	0.11	0.01	-3.46	*Nelumbo nucifera*
CL2073.Contig3_All	1.83	0.91	-1.01	*Vitis vinifera*
Unigene12936_All	2.7	1.35	-1.00	*Eucalyptus grandis*
CL4830.Contig2_All	0.74	1.49	1.01	*Vitis vinifera*
CL4075.Contig3_All	0.53	1.14	1.10	*Vitis vinifera*
Unigene2959_All	1.83	3.98	1.12	*Solanum tuberosum*
CL1604.Contig2_All	1.36	3.11	1.19	*Solanum tuberosum*
Unigene15443_All	0.24	0.73	1.60	*Morus notabilis*
CL4075.Contig2_All	0.57	2.47	2.12	*Nelumbo nucifera*
CL3685.Contig2_All	0.17	2.73	4.01	*Ricinus communis*
CL6747.Contig1_All	4.47	8.94	1.00	*Phoenix dactylifera*
CL2893.Contig4_All	0.85	2.04	1.26	*Eucalyptus grandis*
Unigene13701_All	7.55	20.23	1.42	*Brassica rapa*
CL2893.Contig5_All	0.3	1.22	2.02	*Nelumbo nucifera*

Unigene9831_All	0.57	4.17	2.87	*Nicotianato mentosiformis*
Unigene18733_All	0.55	4.44	3.01	*Theobroma cacao*
Unigene21989_All	0.01	0.23	4.52	*Nelumbo nucifera*
Unigene40680_All	0.01	0.45	5.49	*Triticum urartu*
CL4830.Contig1_All	0.01	1.15	6.85	*Vitis vinifera*
Unigene1080_All	0.01	3.43	8.42	*Solanum lycopersicum*
**WAK**				
Unigene3113_All	2.1	0.01	-7.71	*Eucalyptus grandis*
CL8521.Contig2_All	1.82	0.01	-7.51	*Eucalyptus grandis*
CL1625.Contig3_All	0.67	0.01	-6.07	*Zea mays*
CL219.Contig10_All	4.78	0.16	-4.90	*Citrus sinensis*
CL219.Contig8_All	11.24	2.78	-2.02	*Eucalyptus grandis*
Unigene31535_All	2.38	0.6	-1.99	*Nicotiana sylvestris*
Unigene8224_All	1.73	0.52	-1.73	*Vitis vinifera*
CL219.Contig12_All	9.38	4.19	-1.16	*Eucalyptus grandis*
Unigene34082_All	1.12	0.56	-1.00	*Morus notabilis*
Unigene4677_All	1.58	0.79	-1.00	*Ricinus communis*
Unigene22036_All	1.13	2.49	1.14	*Ricinus communis*
CL2993.Contig1_All	1.12	2.62	1.23	*Eucalyptus grandis*
Unigene4137_All	0.52	1.45	1.48	*Nelumbo nucifera*
Unigene4114_All	1.82	6.37	1.81	*Prunus mume*
Unigene15340_All	0.34	1.35	1.99	*Nicotiana sylvestris*
Unigene850_All	0.01	0.12	3.58	*Ricinus communis*
Unigene36213_All	0.01	0.25	4.64	*Cucumis melo*
Unigene4688_All	0.01	3.04	8.25	*Citrus sinensis*
**LecRLK**				
Unigene12763_All	3.2	0.01	-8.32	*Theobroma cacao*
Unigene22602_All	1.27	0.01	-6.99	*Brassica rapa*
CL2011.Contig2_All	1.27	0.01	-6.99	*Nicotiana sylvestris*
Unigene35797_All	1.16	0.01	-6.86	*Populus trichocarpa*
Unigene37389_All	0.68	0.01	-6.09	*Nicotiana sylvestris*
CL8967.Contig2_All	0.6	0.01	-5.91	*Nicotiana sylvestris*
CL2927.Contig7_All	3.09	0.87	-1.83	*Cucumis sativus*
Unigene7652_All	2.29	0.77	-1.57	*Vitis vinifera*
Unigene16516_All	1.27	0.43	-1.56	*Brassica rapa*
CL865.Contig11_All	1.37	3.13	1.19	*Theobroma cacao*
CL2927.Contig8_All	0.38	0.9	1.24	*Glycine max*
Unigene2748_All	1.35	5	1.89	*Vitis vinifera*
CL3763.Contig2_All	2.05	8.33	2.02	*Nelumbo nucifera*
CL3313.Contig1_All	2.24	11.53	2.36	*Morus notabilis*
Unigene14705_All	0.26	1.54	2.57	*Vitis vinifera*
CL7452.Contig2_All	0.01	0.29	4.86	*Vitis vinifera*
CL865.Contig6_All	0.01	0.38	5.25	*Populus trichocarpa*

CL9743.Contig2_All	0.01	0.66	6.04	*Theobroma cacao*
CL6802.Contig1_All	0.01	1.83	7.52	*Eucalyptus grandis*
Unigene26968_All	0.01	2.43	7.92	*Nicotiana tomentosiformis*
Unigene3683_All	0.01	3.4	8.41	*Solanum lycopersicum*
**LRR-RLK**				
CL608.Contig2_All	2.73	0.01	-8.09	*Atriplex canescens*
CL5423.Contig1_All	1.63	0.01	-7.35	*Nicotiana tomentosiformis*
CL2928.Contig4_All	1.15	0.01	-6.85	*Nelumbo nucifera*
CL6082.Contig1_All	0.77	0.01	-6.27	*Citrus sinensis*
CL1768.Contig5_All	0.76	0.01	-6.25	*Prunus mume*
Unigene25771_All	0.59	0.01	-5.88	*Prunus mume*
Unigene163_All	0.38	0.01	-5.25	*Vitis vinifera*
Unigene29730_All	0.22	0.01	-4.46	*Cucumis sativus*
Unigene29740_All	0.19	0.01	-4.25	*Theobroma cacao*
CL8833.Contig2_All	4.48	0.56	-3.00	*Eucalyptus grandis*
CL6713.Contig1_All	5.91	1.32	-2.16	*Solanum lycopersicum*
Unigene2037_All	0.04	0.01	-2.00	*Theobroma cacao*
CL1231.Contig1_All	4.67	1.41	-1.73	*Theobroma cacao*
Unigene35817_All	4.34	1.36	-1.67	*Vitis vinifera*
Unigene11412_All	1.51	0.65	-1.22	*Pyrus x bretschneideri*
Unigene26697_All	2.76	1.19	-1.21	*Vitis vinifera*
Unigene11127_All	1.3	0.58	-1.16	*Theobroma cacao*
Unigene9888_All	0.94	1.92	1.03	*Nelumbo nucifera*
CL7892.Contig2_All	2.26	5.17	1.19	*Prunus mume*
CL1803.Contig2_All	5.32	12.35	1.22	*Nelumbo nucifera*
CL2084.Contig1_All	2.73	6.76	1.31	*Glycine max*
CL1803.Contig3_All	7.1	19.86	1.48	*Nelumbo nucifera*
Unigene10870_All	0.03	0.09	1.58	*Morus notabilis*
CL1768.Contig6_All	0.13	0.44	1.76	*Vitis vinifera*
CL1729.Contig4_All	3.03	11.55	1.93	*Vitis vinifera*
Unigene3742_All	0.64	2.5	1.97	*Vitis vinifera*
CL7967.Contig2_All	3.99	16.49	2.05	*Nicotiana sylvestris*
Unigene12994_All	2.24	9.49	2.08	*Citrus sinensis*
Unigene39769_All	0.12	0.84	2.81	*Vitis vinifera*
Unigene9196_All	2.96	20.8	2.81	*Citrus sinensis*
CL8833.Contig1_All	0.52	3.94	2.92	*Citrus sinensis*
Unigene5596_All	9.7	82.31	3.09	*Fragaria vesca subsp. vesca*
CL1729.Contig1_All	0.33	6.01	4.19	*Vitis vinifera*
Unigene12746_All	0.05	0.1	1.00	*Pyrus x bretschneideri*
CL8233.Contig2_All	1.94	3.92	1.01	*Vitis vinifera*
CL1803.Contig5_All	15.01	30.8	1.04	*Malus domestica*
Unigene7620_All	1.59	3.41	1.10	*Morus notabilis*
CL5423.Contig2_All	2.96	7.02	1.25	*Nicotiana tomentosiformis*
CL2928.Contig5_All	1.08	2.65	1.29	*Nelumbo nucifera*

CL9137.Contig2_All	5.36	21.52	2.01	*Vitis vinifera*
Unigene38560_All	0.38	1.89	2.31	*Cicer arietinum*
CL1803.Contig1_All	12.83	74.25	2.53	*Eucalyptus grandis*
CL1803.Contig4_All	3.15	63.23	4.33	*Eucalyptus grandis*
CL8471.Contig1_All	0.01	0.15	3.91	*Theobroma cacao*
Unigene39444_All	0.01	0.59	5.88	*Phoenix dactylifera*
CL7338.Contig1_All	0.01	4.2	8.71	*Silene latifolia*
CL2166.Contig2_All	0.01	0.11	3.46	*Solanum tuberosum*
Unigene30007_All	0.01	0.25	4.64	*Pyrus x bretschneideri*
Unigene35311_All	0.01	0.42	5.39	*Pyrus x bretschneideri*
Unigene31462_All	0.01	0.63	5.98	*Phoenix dactylifera*
**LysM**				
Unigene40057_All	0.01	1.01	6.66	*Pyrus x bretschneideri*
CL6066.Contig2_All	0.55	0.01	-5.78	*Nelumbo nucifera*
**CDPK**				
CL280.Contig5_All	0.68	0.01	-6.09	*Theobroma cacao*
CL1897.Contig1_All	0.63	0.01	-5.98	*Nelumbo nucifera*
Unigene4260_All	0.17	0.53	1.64	*Theobroma cacao*
CL6327.Contig1_All	3.96	10.01	1.34	*Theobroma cacao*
CL8340.Contig2_All	0.01	0.22	4.46	*Malus domestica*
**MAPK**				
Unigene30352_All	0.52	0.01	-5.70	*Fragaria vesca subsp. vesca*
Unigene16491_All	9.88	4.05	-1.29	*Erythranthe guttata*
Unigene10295_All	0.65	0.28	-1.22	*Phaseolus vulgaris*
CL578.Contig3_All	3.29	1.49	-1.14	*Citrus sinensis*
Unigene543_All	1.43	0.71	-1.01	
CL9352.Contig2_All	1.04	2.34	1.17	*Erythranthe guttata*
CL1892.Contig2_All	4.93	11.74	1.25	*Vitis vinifera*
CL578.Contig1_All	0.48	1.3	1.44	*Citrus sinensis*
CL4776.Contig1_All	0.01	0.78	6.29	*Vitis vinifera*
CL7688.Contig1_All	0.01	3.84	8.58	*Vitis vinifera*
**CTR**				
CL4515.Contig1_All	0.9	0.38	-1.24	*Vitis vinifera*
**casein**				
CL2804.Contig1_All	4.62	0.01	-8.85	*Vitis vinifera*
CL2804.Contig2_All	2.58	0.01	-8.01	*Vitis vinifera*
CL2804.Contig13_All	1.17	0.01	-6.87	*Vitis vinifera*
CL414.Contig3_All	3.97	1.36	-1.55	*Vitis vinifera*
CL2804.Contig10_All	1.42	10.03	2.82	*Vitis vinifera*
**CL2804.Contig3_All**	0.01	6.21	9.28	*Vitis vinifera*

Table S17. Differentially expressed genes (DEGs) related to protein kinase in roots of *A. canescens* after 100 mM NaCl treatment for 6 h. FPKM-CR6 and FPKM-SR6 respectively indicates the FPKM value of a gene in roots under control condition for 6 h and salt treatment for 6 h. Fold change equals to log2 (FPKM-SR6 / FPKM-CR6). Protein refer to the protein

encoded by each DEGs.

Gene ID	FPKM-CR6	FPKM-SR6	Fold change	Homologous species
**RLK**				
Unigene30353_All	2.72	0.01	-8.09	Ricinus communis
CL4830.Contig1_All	0.6	0.01	-5.91	Vitis vinifera
CL4075.Contig1_All	0.81	0.28	-1.53	Solanum tuberosum
CL3685.Contig2_All	5.02	1.93	-1.38	Ricinus communis
CL7493.Contig1_All	0.77	0.01	-6.27	Nelumbo nucifera
Unigene31860_All	4.55	0.58	-2.97	Eucalyptus grandis
Unigene603_All	4.12	1.27	-1.70	Theobroma cacao
CL2170.Contig4_All	2.6	1.24	-1.07	Prunus mume
CL9166.Contig2_All	2.24	1.07	-1.07	Citrus sinensis
Unigene40582_All	0.49	1.01	1.04	Prunus mume
Unigene10117_All	0.9	2.45	1.44	Theobroma cacao
Unigene35208_All	0.58	4.19	2.85	Vitis vinifera
Unigene1080_All	0.55	4.81	3.13	Solanum lycopersicum
Unigene34019_All	2.2	5.64	1.36	Vitis vinifera
Unigene40680_All	0.47	1.45	1.63	Triticum urartu
CL4830.Contig2_All	0.01	0.53	5.73	Vitis vinifera
Unigene38876_All	0.01	1.14	6.83	Nelumbo nucifera
**WAK**				
CL8521.Contig2_All	3.01	0.01	-8.23	Eucalyptus grandis
CL8953.Contig1_All	0.79	0.01	-6.30	Eucalyptus grandis
CL342.Contig3_All	0.69	0.01	-6.11	Theobroma cacao
Unigene4688_All	0.64	0.01	-6.00	Citrus sinensis
Unigene29472_All	0.61	0.01	-5.93	Nicotiana sylvestris
Unigene4677_All	0.28	0.01	-4.81	Ricinus communis
Unigene22036_All	0.24	0.01	-4.58	Ricinus communis
Unigene8224_All	0.54	0.19	-1.51	Vitis vinifera
CL4189.Contig1_All	1.92	0.79	-1.28	Vitis vinifera
Unigene850_All	3.12	1.34	-1.22	Ricinus communis
CL219.Contig4_All	3.48	7.03	1.01	Nicotiana sylvestris
Unigene36213_All	0.51	1.06	1.06	Cucumis melo
CL219.Contig11_All	1.75	3.7	1.08	Nicotiana sylvestris
CL8521.Contig1_All	1.42	4.39	1.63	Eucalyptus grandis
CL1625.Contig4_All	1.31	4.7	1.84	Solanum lycopersicum
Unigene31535_All	0.62	2.56	2.05	Nicotiana sylvestris
CL7712.Contig2_All	0.01	0.47	5.55	Cucumis sativus
Unigene34082_All	0.01	0.6	5.91	Morus notabilis
Unigene3113_All	0.01	0.75	6.23	Eucalyptus grandis
Unigene36859_All	0.01	1.1	6.78	Solanum lycopersicum
Unigene23885_All	0.01	1.13	6.82	Fragaria vesca subsp. vesca
**LecRLK**				

CL3763.Contig2_All	4.97	0.01	-8.96	Nelumbo nucifera
Unigene35732_All	0.56	0.01	-5.81	Fragaria vesca
Unigene12763_All	4.12	0.11	-5.23	Theobroma cacao
Unigene16277_All	0.15	0.01	-3.91	Cicer arietinum
Unigene9770_All	0.14	0.01	-3.81	Prunus mume
Unigene34574_All	3.33	0.68	-2.29	Citrus sinensis
CL2011.Contig2_All	4.71	1.28	-1.88	Nicotiana sylvestris
CL3313.Contig6_All	1.15	0.39	-1.56	Malus domestica
CL1876.Contig5_All	1.04	0.45	-1.21	Vitis vinifera
Unigene16516_All	4.01	1.98	-1.02	Brassica rapa
CL5713.Contig2_All	1.79	3.81	1.09	Malus domestica
CL3763.Contig1_All	3.61	10.17	1.49	Nelumbo nucifera
Unigene38082_All	0.69	2.11	1.61	Zea mays
CL1044.Contig1_All	1.15	3.55	1.63	Cucumis melo
Unigene35797_All	0.7	2.99	2.09	Populus trichocarpa
CL2927.Contig7_All	1.36	7.04	2.37	Cucumis sativus
Unigene3683_All	0.19	3.22	4.08	Solanum lycopersicum
CL30.Contig6_All	0.01	0.36	5.17	Nicotiana tomentosiformis
Unigene2748_All	0.01	0.73	6.19	Vitis vinifera
CL4407.Contig2_All	0.01	1.49	7.22	Prunus mume
CL9743.Contig2_All	0.01	2.11	7.72	Theobroma cacao
**LRR-RLK**				
Unigene32199_All	2.24	0.01	-7.81	Setaria italica
Unigene31458_All	2.18	0.01	-7.77	Eucalyptus grandis
CL7338.Contig2_All	1.66	0.01	-7.38	Dimocarpus longan
Unigene25771_All	1.24	0.01	-6.95	Prunus mume
CL5636.Contig2_All	0.31	0.01	-4.95	Citrus sinensis
CL1803.Contig3_All	0.21	0.01	-4.39	Nelumbo nucifera
CL1768.Contig9_All	3.12	0.61	-2.35	Prunus mume
Unigene39769_All	4.38	0.9	-2.28	Vitis vinifera
CL9025.Contig1_All	7.6	1.77	-2.10	Cicer arietinum
CL371.Contig2_All	2.83	0.86	-1.72	Malus domestica
Unigene33825_All	1.24	0.42	-1.56	Pyrus x bretschneideri
Unigene31722_All	1.8	0.61	-1.56	Citrus sinensis
Unigene12747_All	5.46	1.96	-1.48	Nicotiana tomentosiformis
CL608.Contig2_All	4.89	2.38	-1.04	Atriplex canescens
CL371.Contig3_All	1.23	2.5	1.02	Prunus mume
Unigene39549_All	0.94	1.92	1.03	Vitis vinifera
Unigene30665_All	0.32	0.66	1.04	Fragaria vesca subsp. vesca
CL1768.Contig5_All	0.51	1.07	1.07	Prunus mume
Unigene2009_All	1.74	4.43	1.35	Vitis vinifera
Unigene12746_All	2.97	7.58	1.35	Pyrus x bretschneideri
CL8233.Contig2_All	0.94	2.68	1.51	Vitis vinifera
CL7338.Contig1_All	0.49	3	2.61	Silene latifolia

CL1803.Contig2_All	0.01	0.19	4.25	Nelumbo nucifera
Unigene13207_All	0.01	0.34	5.09	Vitis vinifera
Unigene2487_All	0.01	0.41	5.36	Eucalyptus grandis
CL5423.Contig4_All	0.01	0.55	5.78	Eucalyptus grandis
CL1231.Contig3_All	0.01	0.59	5.88	Theobroma cacao
Unigene39444_All	0.01	1.28	7.00	Phoenix dactylifera
**LysM**				
Unigene28415_All	1.77	3.54	1.00	Cercis chinensis
**CDPK**				
CL3423.Contig2_All	2.19	0.01	-7.77	Chenopodium album
CL9501.Contig1_All	0.9	0.01	-6.49	Nicotiana tomentosiformis
CL3423.Contig1_All	1.87	3.76	1.01	Chenopodium album
CL6327.Contig1_All	5.98	12.03	1.01	Theobroma cacao
Unigene4260_All	0.92	2.07	1.17	Theobroma cacao
CL280.Contig8_All	1.15	2.68	1.22	Theobroma cacao
Unigene36941_All	1.63	4.52	1.47	Populus trichocarpa
CL280.Contig5_All	0.01	0.03	1.58	Theobroma cacao
CL3423.Contig3_All	0.01	0.2	4.32	Chenopodium album
**MAPK**				
Unigene543_All	9.29	1.54	-2.59	
CL578.Contig2_All	1.38	0.53	-1.38	Theobroma cacao
Unigene30352_All	0.55	1.12	1.03	Fragaria vesca subsp. vesca
CL8162.Contig2_All	0.5	1.3	1.38	Citrus clementina
CL7688.Contig1_All	3.03	8.27	1.45	Vitis vinifera
Unigene6293_All	0.39	1.12	1.52	Solanum lycopersicum
CL1892.Contig1_All	0.1	0.53	2.41	Vitis vinifera
CL9694.Contig2_All	0.01	1.19	6.89	Vitis vinifera
CL1892.Contig2_All	0.01	1.33	7.06	Vitis vinifera
**casein kinase**				
CL2804.Contig3_All	5.11	0.01	-9.00	Vitis vinifera
CL2804.Contig12_All	0.67	0.01	-6.07	Vitis vinifera

Table S18. Differentially expressed genes (DEGs) related to protein kinase in roots of *A. canescens* after 100 mM NaCl treatment for 24 h. FPKM-CR24 and FPKM-SR24 respectively indicates the FPKM value of a gene in roots under control condition for 24 h and salt treatment for 24 h. Fold change equals to log2 (FPKM-SR24 / FPKM-SR24). Protein refer to the protein encoded by each DEGs.

Gene ID	FPKM-CR24	FPKM-SR24	Fold change	Homologous species
**RLK**				
Unigene30353_All	2.7	0.01	-8.08	*Ricinus communis*
Unigene36621_All	1.82	0.01	-7.51	*Eucalyptus grandis*
Unigene15443_All	0.5	0.01	-5.64	*Morus notabilis*

Unigene12732_All	7.05	0.19	-5.21	*Ricinus communis*
Unigene2959_All	6.02	1.54	-1.97	*Solanum tuberosum*
Unigene40582_All	3.41	0.95	-1.84	*Prunus mume*
CL4075.Contig3_All	4.27	1.34	-1.67	*Vitis vinifera*
CL1604.Contig1_All	17.68	5.99	-1.56	*Vitis vinifera*
Unigene7507_All	2.18	0.76	-1.52	*Morus notabilis*
CL1604.Contig3_All	9.92	3.56	-1.48	*Vitis vinifera*
Unigene11654_All	31	14.89	-1.06	*Nicotiana sylvestris*
Unigene10117_All	1.18	0.57	-1.05	*Theobroma cacao*
CL3685.Contig2_All	3.91	1.89	-1.05	*Ricinus communis*
CL1604.Contig2_All	1.82	0.88	-1.05	*Solanum tuberosum*
Unigene13978_All	85.36	0.08	-10.06	*Pyrus x bretschneideri*
CL2170.Contig5_All	5.99	0.01	-9.23	*Nelumbo nucifera*
Unigene616_All	2.59	0.01	-8.02	*Theobroma cacao*
CL3305.Contig2_All	1.48	0.01	-7.21	*Nelumbo nucifera*
CL7493.Contig1_All	1.45	0.01	-7.18	*Nelumbo nucifera*
CL7451.Contig5_All	1.36	0.01	-7.09	*Phoenix dactylifera*
Unigene33490_All	1.27	0.01	-6.99	*Pyrus x bretschneideri*
CL5638.Contig4_All	1.04	0.01	-6.70	*Vitis vinifera*
Unigene36094_All	0.9	0.01	-6.49	*Nelumbo nucifera*
CL2170.Contig3_All	0.77	0.01	-6.27	*Morus notabilis*
Unigene9831_All	3.72	0.19	-4.29	*Nicotiana tomentosiformis*
CL7493.Contig2_All	27.06	2.23	-3.60	*Nelumbo nucifera*
Unigene15867_All	6.97	0.59	-3.56	*Vitis vinifera*
Unigene34017_All	1.91	0.22	-3.12	*Nelumbo nucifera*
CL2170.Contig4_All	3.33	0.63	-2.40	*Prunus mume*
CL2073.Contig3_All	3.64	0.77	-2.24	*Vitis vinifera*
Unigene29556_All	2.27	0.49	-2.21	*Malus domestica*
CL6747.Contig1_All	6.88	1.78	-1.95	*Phoenix dactylifera*
Unigene17553_All	13.41	3.52	-1.93	*Prunus mume*
Unigene30033_All	3.63	1.06	-1.78	*Musa acuminata subsp. malaccensis*
Unigene12936_All	2.93	0.99	-1.57	*Eucalyptus grandis*
CL264.Contig1_All	59.77	21.55	-1.47	*Vitis vinifera*
Unigene16763_All	11.85	4.46	-1.41	*Prunus mume*
CL2073.Contig1_All	3.46	1.37	-1.34	*Vitis vinifera*
Unigene3618_All	99.71	46.46	-1.10	*Vitis vinifera*
Unigene18733_All	1.15	0.56	-1.04	*Theobroma cacao*
Unigene21989_All	2.39	1.18	-1.02	*Nelumbo nucifera*
CL6175.Contig1_All	2.22	5.99	1.43	*Vitis vinifera*
Unigene39347_All	0.93	2.71	1.54	*Nelumbo nucifera*
Unigene13701_All	1.69	19.9	3.56	*Brassica rapa*
CL1768.Contig7_All	0.31	5.68	4.20	*Prunus mume*
Unigene14535_All	0.31	5.99	4.27	*Theobroma cacao*
CL4075.Contig1_All	0.53	1.29	1.28	*Solanum tuberosum*

Unigene16773_All	2.23	7.66	1.78	*Theobroma cacao*
Unigene39455_All	0.01	0.64	6.00	*Arabidopsis thaliana*
**WAK**				
CL171.Contig1_All	3.8	0.01	-8.57	*Medicago truncatula*
Unigene36213_All	3.06	0.01	-8.26	*Cucumis melo*
Unigene35516_All	2.48	0.01	-7.95	*Nelumbo nucifera*
Unigene14495_All	2.34	0.01	-7.87	*Citrus sinensis*
CL8953.Contig1_All	2.34	0.01	-7.87	*Eucalyptus grandis*
CL1625.Contig3_All	1.38	0.01	-7.11	*Zea mays*
Unigene31535_All	1.24	0.01	-6.95	*Nicotiana sylvestris*
Unigene40387_All	1.23	0.01	-6.94	*Zea mays*
Unigene23885_All	1.09	0.01	-6.77	*Fragaria vesca subsp. vesca*
Unigene36859_All	1.06	0.01	-6.73	*Solanum lycopersicum*
Unigene39008_All	0.67	0.01	-6.07	*Nelumbo nucifera*
CL7712.Contig3_All	15.73	0.24	-6.03	*Nelumbo nucifera*
Unigene29472_All	0.61	0.01	-5.93	*Nicotiana sylvestris*
Unigene34082_All	0.58	0.01	-5.86	*Morus notabilis*
CL7712.Contig1_All	14.49	0.4	-5.18	*Nelumbo nucifera*
Unigene16011_All	24.73	0.69	-5.16	*Nelumbo nucifera*
Unigene29407_All	8.2	0.39	-4.39	*Vitis vinifera*
CL6066.Contig1_All	7.35	0.42	-4.13	*Ricinus communis*
CL1625.Contig1_All	4.88	0.63	-2.95	*Nelumbo nucifera*
Unigene850_All	2.7	0.37	-2.87	*Ricinus communis*
Unigene15340_All	5.59	1.02	-2.45	*Nicotiana sylvestris*
CL2993.Contig2_All	1.99	0.41	-2.28	*Fragaria vesca subsp. vesca*
CL219.Contig7_All	6.04	1.95	-1.63	*Nelumbo nucifera*
CL8521.Contig2_All	3.01	1.18	-1.35	*Eucalyptus grandis*
Unigene5171_All	191.89	90.55	-1.08	*Nicotiana sylvestris*
CL2993.Contig1_All	0.77	1.95	1.34	*Eucalyptus grandis*
Unigene4114_All	0.86	2.83	1.72	*Prunus mume*
Unigene20305_All	0.33	3.22	3.29	*Citrus sinensis*
Unigene8224_All	0.18	3.13	4.12	*Vitis vinifera*
CL7712.Contig2_All	0.08	2.22	4.79	*Cucumis sativus*
CL342.Contig3_All	0.01	0.66	6.04	*Theobroma cacao*
CL219.Contig10_All	0.01	1.13	6.82	*Citrus sinensis*
Unigene22036_All	0.01	2.05	7.68	*Ricinus communis*
Unigene4677_All	0.01	2.13	7.73	*Ricinus communis*
Unigene2643_All	0.01	5.09	8.99	*Theobroma cacao*
**LecRLK**				
CL2011.Contig2_All	10.71	0.01	-10.06	*Nicotiana sylvestris*
CL3313.Contig6_All	4.94	0.01	-8.95	*Malus domestica*
CL9743.Contig2_All	2.71	0.01	-8.08	*Theobroma cacao*
Unigene20530_All	1.64	0.01	-7.36	*Theobroma cacao*

Unigene22602_All	1.32	0.01	-7.04	*Brassica rapa*
Unigene34574_All	1.32	0.01	-7.04	*Citrus sinensis*
CL6802.Contig1_All	1.26	0.01	-6.98	*Eucalyptus grandis*
Unigene40630_All	1.15	0.01	-6.85	*Theobroma cacao*
Unigene18214_All	31.98	0.6	-5.74	*Eucalyptus grandis*
CL1876.Contig4_All	30.03	0.81	-5.21	*Vitis vinifera*
CL1876.Contig1_All	12.49	0.34	-5.20	*Vitis vinifera*
Unigene40469_All	0.35	0.01	-5.13	*Populus trichocarpa*
CL389.Contig2_All	7.62	0.63	-3.60	*Vitis vinifera*
Unigene26968_All	3.02	0.34	-3.15	*Nicotiana tomentosiformis*
CL2011.Contig1_All	18.29	2.1	-3.12	*Citrus sinensis*
Unigene29873_All	1.19	0.14	-3.09	*Theobroma cacao*
CL1044.Contig1_All	3.43	0.55	-2.64	*Cucumis melo*
CL3313.Contig1_All	26.53	4.29	-2.63	*Morus notabilis*
CL8882.Contig1_All	8.44	1.39	-2.60	*Citrus sinensis*
Unigene3944_All	2.95	0.54	-2.45	*Vitis vinifera*
Unigene7995_All	2.44	0.47	-2.38	*Nelumbo nucifera*
CL1876.Contig2_All	12.89	2.53	-2.35	*Vitis vinifera*
Unigene12173_All	9.06	2.05	-2.14	*Nelumbo nucifera*
Unigene14705_All	1.96	0.46	-2.09	*Vitis vinifera*
CL2927.Contig1_All	8.98	2.18	-2.04	*Cucumis sativus*
Unigene35732_All	2.21	0.54	-2.03	*Fragaria vesca subsp. vesca*
CL7452.Contig2_All	2.13	0.55	-1.95	*Vitis vinifera*
CL232.Contig6_All	7.3	2.34	-1.64	*Morus notabilis*
CL136.Contig1_All	9.28	2.99	-1.63	*Nicotiana tomentosiformis*
CL3763.Contig3_All	2.31	0.76	-1.60	*Nelumbo nucifera*
Unigene14931_All	4.04	1.51	-1.42	*Prunus mume*
CL30.Contig9_All	3.1	1.17	-1.41	*Eucalyptus grandis*
CL2927.Contig2_All	6.87	2.62	-1.39	*Cucumis sativus*
CL5713.Contig2_All	3.39	1.3	-1.38	*Malus domestica*
CL3763.Contig1_All	7.53	3.11	-1.28	*Nelumbo nucifera*
CL2927.Contig4_All	7.46	3.12	-1.26	*Cucumis sativus*
Unigene16894_All	26.1	11.01	-1.25	*Theobroma cacao*
Unigene12686_All	2.59	1.13	-1.20	*Vitis vinifera*
Unigene27493_All	2.1	1.02	-1.04	*Vitis vinifera*
Unigene114_All	1.56	0.76	-1.04	*Vitis vinifera*
CL1876.Contig5_All	0.92	1.94	1.08	*Vitis vinifera*
Unigene7652_All	1.06	2.57	1.28	*Vitis vinifera*
CL2927.Contig7_All	0.62	1.81	1.55	*Cucumis sativus*
CL2927.Contig3_All	1.36	4.03	1.57	*Theobroma cacao*
CL1044.Contig2_All	1.12	4.1	1.87	*Vitis vinifera*
Unigene37389_All	0.35	1.36	1.96	*Nicotiana sylvestris*
Unigene2748_All	0.28	1.09	1.96	*Vitis vinifera*
CL4407.Contig1_All	1.61	9.13	2.50	*Malus domestica*

CL8967.Contig2_All	0.31	1.8	2.54	*Nicotiana sylvestris*
CL865.Contig11_All	0.17	3.72	4.45	*Theobroma cacao*
CL7244.Contig1_All	0.01	0.58	5.86	*Vitis vinifera*
CL3763.Contig2_All	0.01	0.64	6.00	*Nelumbo nucifera*
CL4407.Contig2_All	0.01	0.89	6.48	*Prunus mume*
CL7244.Contig2_All	0.01	2.32	7.86	*Nelumbo nucifera*
CL9096.Contig2_All	0.01	3.18	8.31	*Vitis vinifera*
Unigene9770_All	0.01	6.98	9.45	*Prunus mume*
Unigene16277_All	0.01	19.63	10.94	*Cicer arietinum*
**LRR-RLK**				
CL1483.Contig1_All	19.47	4.68	-2.06	*Nicotiana sylvestris*
CL1729.Contig1_All	1.3	0.49	-1.41	*Vitis vinifera*
CL1729.Contig3_All	0.79	0.01	-6.30	*Musa acuminata subsp. malaccensis*
CL1729.Contig4_All	4.51	1.16	-1.96	*Vitis vinifera*
CL1768.Contig6_All	2.74	0.01	-8.10	*Vitis vinifera*
CL1768.Contig9_All	2	0.51	-1.97	*Prunus mume*
CL1909.Contig2_All	8.97	3.72	-1.27	*Nicotiana tomentosiformis*
CL2084.Contig1_All	116.75	3.11	-5.23	*Glycine max*
CL2166.Contig2_All	27.16	0.01	-11.41	*Solanum tuberosum*
CL371.Contig2_All	2.22	0.34	-2.71	*Malus domestica*
CL371.Contig3_All	2.65	0.01	-8.05	*Prunus mume*
CL4321.Contig1_All	28.78	10.11	-1.51	*Vitis vinifera*
CL4514.Contig2_All	18.08	5.48	-1.72	*Solanum lycopersicum*
CL4705.Contig1_All	13.01	3.45	-1.91	*Vitis vinifera*
CL5636.Contig2_All	0.26	0.01	-4.70	*Citrus sinensis*
CL6082.Contig1_All	0.42	0.01	-5.39	*Citrus sinensis*
CL652.Contig2_All	19.06	7.31	-1.38	*Nelumbo nucifera*
CL6713.Contig3_All	15.21	5.25	-1.53	*Vitis vinifera*
CL7365.Contig2_All	6.23	1.99	-1.65	*Vitis vinifera*
CL7879.Contig1_All	18.18	5.57	-1.71	*Prunus mume*
CL7879.Contig2_All	26.14	6.71	-1.96	*Prunus mume*
CL7881.Contig2_All	11.66	1.16	-3.33	*Pyrus x bretschneideri*
CL7892.Contig2_All	6.95	2.49	-1.48	*Prunus mume*
CL9025.Contig1_All	2.69	1.28	-1.07	*Cicer arietinum*
CL974.Contig2_All	8.5	0.91	-3.22	*Citrus sinensis*
Unigene10870_All	3.76	0.03	-6.97	*Morus notabilis*
Unigene12036_All	61.3	0.01	-12.58	*Fragaria vesca subsp. vesca*
Unigene12994_All	4.46	1.21	-1.88	*Citrus sinensis*
Unigene13054_All	4.26	1.44	-1.56	*Vitis vinifera*
Unigene14583_All	6.58	0.01	-9.36	*Malus domestica*
Unigene1518_All	20.9	5.36	-1.96	*Malus domestica*
Unigene16745_All	33.27	15.56	-1.10	*Theobroma cacao*
Unigene1911_All	6.26	0.08	-6.29	*Prunus mume*
Unigene2462_All	98.07	29.77	-1.72	*Prunus mume*

Unigene27010_All	1.51	0.75	-1.01	*Malus domestica*
Unigene30665_All	0.96	0.01	-6.58	*Fragaria vesca subsp. vesca*
Unigene31458_All	2.15	0.01	-7.75	*Eucalyptus grandis*
Unigene3321_All	13.42	3.34	-2.01	*Vitis vinifera*
Unigene33825_All	2.04	0.79	-1.37	*Pyrus x bretschneideri*
Unigene35162_All	1.1	0.01	-6.78	*Eucalyptus grandis*
Unigene35960_All	6.18	0.01	-9.27	*Fragaria vesca subsp. vesca*
Unigene37147_All	4.29	0.01	-8.74	*Nicotiana tomentosiformis*
Unigene39549_All	1.39	0.01	-7.12	*Vitis vinifera*
Unigene39769_All	4.83	0.48	-3.33	*Vitis vinifera*
Unigene4286_All	8.02	1.67	-2.26	*Vitis vinifera*
Unigene4401_All	25.84	8.48	-1.61	*Vitis vinifera*
Unigene450_All	6.96	0.01	-9.44	*Nelumbo nucifera*
Unigene5596_All	187.25	65.7	-1.51	*Fragaria vesca subsp. vesca*
Unigene6674_All	14.49	2.93	-2.31	*Prunus mume*
Unigene9888_All	1.69	0.63	-1.42	*Nelumbo nucifera*
CL2084.Contig2_All	127.68	4.8	-4.73	*Vitis vinifera*
CL2166.Contig1_All	34.41	0.01	-11.75	*Vitis vinifera*
CL2166.Contig3_All	20.79	0.01	-11.02	*Vitis vinifera*
CL2166.Contig4_All	23.27	0.01	-11.18	*Vitis vinifera*
CL2928.Contig4_All	6.5	0.01	-9.34	*Nelumbo nucifera*
CL2928.Contig5_All	11.53	1.44	-3.00	*Nelumbo nucifera*
CL5423.Contig4_All	0.53	0.01	-5.73	*Eucalyptus grandis*
CL8471.Contig2_All	20.8	6.74	-1.63	*Theobroma cacao*
CL9535.Contig1_All	37.85	15.55	-1.28	*Theobroma cacao*
Unigene10979_All	15.46	2.54	-2.61	*Arabidopsis thaliana*
Unigene11029_All	2.47	0.46	-2.42	*Populus trichocarpa*
Unigene11338_All	3.84	0.65	-2.56	*Pyrus x bretschneideri*
Unigene11487_All	39.09	8.72	-2.16	*Vitis vinifera*
Unigene11535_All	36.34	1.12	-5.02	*Morus notabilis*
Unigene12746_All	3.63	0.01	-8.50	*Pyrus x bretschneideri*
Unigene12747_All	5.46	0.05	-6.77	*Nicotiana tomentosiformis*
Unigene12904_All	8.34	2.14	-1.96	*Vitis vinifera*
Unigene13104_All	5.28	2.63	-1.01	*Theobroma cacao*
Unigene162_All	11.23	0.41	-4.78	*Theobroma cacao*
Unigene163_All	3.17	0.53	-2.58	*Vitis vinifera*
Unigene16398_All	28.49	1.79	-3.99	*Theobroma cacao*
Unigene17179_All	19.6	4.44	-2.14	*Vitis vinifera*
Unigene2037_All	4.32	0.01	-8.75	*Theobroma cacao*
Unigene239_All	17.73	1.18	-3.91	*Nicotiana tomentosiformis*
Unigene29730_All	2.31	0.22	-3.39	*Cucumis sativus*
Unigene29740_All	2.06	0.1	-4.36	*Theobroma cacao*
Unigene2979_All	11.15	4	-1.48	*Vitis vinifera*
Unigene31266_All	1.43	0.01	-7.16	*Ricinus communis*

Unigene36355_All	1.35	0.01	-7.08	*Solanum tuberosum*
Unigene38082_All	1.36	0.01	-7.09	*Zea mays*
Unigene38560_All	1.17	0.01	-6.87	*Cicer arietinum*
Unigene39444_All	1.23	0.01	-6.94	*Phoenix dactylifera*
Unigene4127_All	5.93	2.73	-1.12	*Theobroma cacao*
Unigene5546_All	5.75	2.04	-1.49	*Nicotiana sylvestris*
Unigene5661_All	6.14	0.39	-3.98	*Nelumbo nucifera*
Unigene6058_All	9.98	3.36	-1.57	*Ricinus communis*
Unigene6612_All	19.66	2.42	-3.02	*Theobroma cacao*
Unigene6664_All	16.82	7.14	-1.24	*Citrus sinensis*
Unigene5989_All	4.33	9.33	1.11	*Nicotiana sylvestris*
Unigene11342_All	1.04	2.35	1.18	*Eucalyptus grandis*
CL1768.Contig5_All	0.33	0.84	1.35	*Prunus mume*
Unigene9196_All	1.3	4.09	1.65	*Citrus sinensis*
CL1803.Contig2_All	0.72	10.05	3.80	*Nelumbo nucifera*
CL1803.Contig3_All	0.19	53.68	8.14	*Nelumbo nucifera*
CL7338.Contig2_All	0.55	1.59	1.53	*Dimocarpus longan*
CL9137.Contig2_All	1.35	3.98	1.56	*Vitis vinifera*
CL4352.Contig1_All	6.01	19.47	1.70	*Morus notabilis*
CL5874.Contig2_All	0.96	3.25	1.76	*Theobroma cacao*
Unigene16773_All	2.23	7.66	1.78	*Theobroma cacao*
Unigene2009_All	1.69	6.34	1.91	*Vitis vinifera*
Unigene1046_All	17.21	66.09	1.94	*Vitis vinifera*
CL7338.Contig1_All	0.48	1.87	1.96	*Silene latifolia*
Unigene7620_All	0.94	4.75	2.34	*Morus notabilis*
Unigene15613_All	3.23	17.89	2.47	*Populus trichocarpa*
CL8233.Contig1_All	0.46	2.73	2.57	*Vitis vinifera*
CL5423.Contig2_All	1.44	8.86	2.62	*Nicotiana tomentosiformis*
CL5423.Contig3_All	1.64	14.19	3.11	*Nicotiana tomentosiformis*
Unigene7808_All	0.78	23.89	4.94	*Vitis vinifera*
CL1803.Contig1_All	1.17	48.21	5.36	*Eucalyptus grandis*
CL1803.Contig5_All	0.35	30.97	6.47	*Malus domestica*
CL1231.Contig1_All	0.01	2.56	8.00	*Theobroma cacao*
Unigene26697_All	0.01	3.3	8.37	*Vitis vinifera*
CL1803.Contig4_All	0.01	61.6	12.59	*Eucalyptus grandis*
Unigene35311_All	0.01	0.85	6.41	*Pyrus x bretschneideri*
Unigene32199_All	0.01	1.08	6.75	*Setaria italica*
Unigene35817_All	0.01	1.64	7.36	*Vitis vinifera*
Unigene31462_All	0.01	1.88	7.55	*Phoenix dactylifera*
Unigene13207_All	0.01	2.9	8.18	*Vitis vinifera*
**LysM**				
CL6066.Contig2_All	5.16	0.01	-9.01	*Nelumbo nucifera*
Unigene28415_All	4.25	0.01	-8.73	*Cercis chinensis*
CL4753.Contig2_All	3.77	0.46	-3.03	*Nelumbo nucifera*

Unigene3594_All	6.28	2.99	-1.07	*Nelumbo nucifera*
**CDPK**				
CL8340.Contig1_All	14.93	1	-3.90	*Theobroma cacao*
Unigene9182_All	24.42	3.51	-2.80	*Theobroma cacao*
CL4209.Contig1_All	6.03	0.88	-2.78	*Theobroma cacao*
Unigene18397_All	5.01	0.79	-2.66	*Haloxylon ammodendron*
Unigene2132_All	2.72	0.8	-1.77	*Malus domestica*
Unigene7837_All	60.39	18.57	-1.70	*Vitis vinifera*
Unigene299_All	1.72	0.53	-1.70	*Prunus mume*
Unigene3918_All	1.97	0.64	-1.62	*Brassica rapa*
CL5216.Contig1_All	23.61	7.81	-1.60	*Mesembryanthemum crystallinum*
Unigene36941_All	2.88	1.08	-1.42	*Populus trichocarpa*
CL4396.Contig1_All	28.05	11.19	-1.33	*Theobroma cacao*
CL297.Contig8_All	10.18	4.12	-1.31	*Ricinus communis*
CL4396.Contig2_All	39.52	16.04	-1.30	*Theobroma cacao*
CL2236.Contig3_All	15.03	6.68	-1.17	*Haloxylon ammodendron*
CL2236.Contig4_All	16.73	7.85	-1.09	*Haloxylon ammodendron*
CL280.Contig5_All	1.27	0.62	-1.03	*Theobroma cacao*
CL2236.Contig1_All	10.01	4.9	-1.03	*Haloxylon ammodendron*
CL280.Contig4_All	1.48	3.12	1.08	*Theobroma cacao*
**MAPK**				
CL4776.Contig2_All	3.16	0.01	-8.30	*Vitis vinifera*
Unigene22967_All	41.09	3.97	-3.37	*Coffea canephora*
Unigene10295_All	5.84	0.74	-2.98	*Phaseolus vulgaris*
CL3454.Contig4_All	19.79	2.81	-2.82	*Humulus lupulus*
Unigene6293_All	17.73	2.66	-2.74	*Solanum lycopersicum*
Unigene10696_All	14.21	2.39	-2.57	*Citrus clementina*
CL3454.Contig2_All	82.51	14.81	-2.48	*Arabis alpina*
Unigene543_All	14.46	3.2	-2.18	
CL3454.Contig3_All	141.08	32.93	-2.10	*Humulus lupulus*
CL3454.Contig1_All	120.76	29.21	-2.05	*Humulus lupulus*
Unigene8714_All	49.14	14.35	-1.78	*Medicago truncatula*
Unigene8714_All	49.14	14.35	-1.78	*Medicago truncatula*
Unigene3216_All	46.08	14.29	-1.69	*Prunus persica*
CL9352.Contig2_All	11.11	3.49	-1.67	*Erythranthe guttata*
CL7275.Contig1_All	6.97	2.36	-1.56	*Chenopodium album*
CL3427.Contig1_All	35.9	13.35	-1.43	*Beta vulgaris*
CL7275.Contig2_All	19.06	7.92	-1.27	*Chenopodium album*
CL7275.Contig3_All	26.87	11.28	-1.25	*Chenopodium album*
CL9694.Contig1_All	3.74	1.77	-1.08	*Citrus sinensis*
CL4776.Contig1_All	5.88	2.8	-1.07	*Vitis vinifera*
CL8162.Contig2_All	1.55	0.74	-1.07	*Citrus clementina*
CL7688.Contig1_All	7.15	3.53	-1.02	*Vitis vinifera*

Unigene6939_All	32.08	15.84	-1.02	*Beta vulgaris*
Unigene4202_All	22.55	11.26	-1.00	*Citrus sinensis*
CL7311.Contig3_All	1.02	3.13	1.62	
Unigene14008_All	0.51	7.67	3.91	*Vitis vinifera*
CL1892.Contig1_All	0.2	29.67	7.21	*Vitis vinifera*
CL578.Contig2_All	0.01	1.93	7.59	*Theobroma cacao*
CL1892.Contig2_All	0.01	2.48	7.95	*Vitis vinifera*
**CTR1**				
CL9787.Contig2_All	8.61	60.56	2.81	*Celosia argentea*
CL4515.Contig1_All	8.91	1.03	-3.11	*Vitis vinifera*
CL4515.Contig2_All	10.13	1.3	-2.96	*Vitis vinifera*
CL6621.Contig2_All	22.78	11.07	-1.04	*Vitis vinifera*
casein				
CL2804.Contig3_All	0.01	1.24	6.95	*Vitis vinifera*
CL2804.Contig12_All	0.31	0.01	-4.95	*Vitis vinifera*
CL2804.Contig4_All	5.61	1.07	-2.39	*Vitis vinifera*
Unigene2822_All	4.47	1.13	-1.98	*Morus notabilis*
Unigene3220_All	14.63	6.31	-1.21	*Theobroma cacao*
Unigene15130_All	38.83	17.38	-1.16	*Beta vulgaris*

Table S19 Expression pattern validation of 7 randomly selected genes in in leaves and roots of *A. canescens* under 100 mM NaCl treatment for 6 h by qRT-PCR.

Gene ID	Leaf		Root	
	Log2 Ratio (SL/CL)		Log2 Ratio (SR/CR)	
	RNA-seq	qPCR	RNA-seq	qPCR
CL9208.Contig1_All	3.60	4.82	-3.17	-1.76
CL1844.Contig2_All	5.51	4.87	-6.99	-4.68
Unigene9476_All	6.23	7.29	-7.25	-4.83
Unigene5013_All	14.63	9.67	-11.38	-10.56
CL3665.Contig1_All	9.82	8.88	-9.60	-7.98
CL2142.Contig5_All	-3.04	-4.13	1.89	3.64
CL1382.Contig4_All	7.21	7.41	-14.57	-8.52

Table S20 Primer sequences used in qRT-PCR

Primers	Sequences (5′-3′)
CL9208.Contig1_All-F	CCAAGAACTCAATCACTATC
CL9208.Contig1_All-R	TCCAAGATTACTACCCATAC
CL1844.Contig2_All-F	TCCTAGAGAACACATGATGA
CL1844.Contig2_All-R	CGGCGAGAAGTGATTATG
Unigene9476_All-F	CTAACCGAGACATTAACCA
Unigene9476_All-R	GTTGAGATACCTTGAACTTG

Unigene5013_All-F	CGAGCCACTGAGATATTC
Unigene5013_All-R	AAATGTATGAGACCCTGAG
CL3665.Contig1_All-F	TACACGGATTCAAGGACAA
CL3665.Contig1_All-R	CACTTCCACCACAGACTT
CL2142.Contig5_All-F	GGGCACATACTCTTGGAA
CL2142.Contig5_All-R	CCATCTTAGGCATTCATCATC
CL1382.Contig4_All-F	AGCCTCTAAACTCAACTATG
CL1382.Contig4_All-R	GTAGATCAGCCAAAGTGTA
Actin-F	AAGAACTACGAGCTACCTGACGG
Actin-R	GATACCAGAAGATTCCATTCCAAC
